# Intercellular transfer of exosomal wild type EGFR triggers osimertinib resistance in non-small cell lung cancer

**DOI:** 10.1186/s12943-021-01307-9

**Published:** 2021-01-18

**Authors:** Shaocong Wu, Min Luo, Kenneth K. W. To, Jianye Zhang, Chaoyue Su, Hong Zhang, Sainan An, Fang Wang, Da Chen, Liwu Fu

**Affiliations:** 1 State Key Laboratory of Oncology in South China, Collaborative Innovation Center for Cancer Medicine; Guangdong Esophageal Cancer Institute; Sun Yat-sen University Cancer Center, Guangzhou, 510060 People’s Republic of China; 2 School of Pharmacy, Faculty of Medicine, The Chinese University of Hong Kong, Room 801N, Area 39, Lo Kwee-Seong Integrated Biomedical Sciences Building, Shatin, New Territories, Hong Kong, SAR China; 3 School of Pharmaceutical Sciences, Guangzhou Medical University, Guangzhou, 511436 China

**Keywords:** Wild type EGFR, Exosomes, Osimertinib, NSCLC, Acquired resistance

## Abstract

**Background:**

Epidermal growth factor receptor (EGFR)-mutated lung cancer constitutes a major subgroup of non-small cell lung cancer (NSCLC) and osimertinib is administrated as first-line treatment. However, most patients with osimertinib treatment eventually relapse within one year. The underlying mechanisms of osimertinib resistance remain largely unexplored.

**Methods:**

Exosomes isolation was performed by differential centrifugation. Co-culture assays were conducted to explore the alteration of drug sensitivity by cell viability and apoptosis assays. Immunofluorescence and flow cytometry were performed to visualize the formation or absorption of exosomes. Exosomes secretion was measured by Nanoparticle Tracking Analysis or ELISA. The xenograft tumor model in mice was established to evaluate the effect of exosomes on osimertinib sensitivity in vivo.

**Results:**

Intercellular transfer of exosomal wild type EGFR protein confers osimertinib resistance to EGFR-mutated sensitive cancer cells in vitro and in vivo. Co-culture of EGFR-mutated sensitive cells and EGFR-nonmutated resistant cells promoted osimertinib resistance phenotype in EGFR-mutated cancer cells, while depletion of exosomes from conditioned medium or blockade of exosomal EGFR by neutralizing antibody alleviated this phenotype. Mechanistically, osimertinib promoted the release of exosomes by upregulated a Rab GTPase (RAB17). Knockdown of RAB17 resulted in the decrease of exosomes secretion. Moreover, exosomes could be internalized by EGFR-mutated cancer cells via Clathrin-dependent endocytosis and then the encapsulated exosomal wild type EGFR protein activated downstream PI3K/AKT and MAPK signaling pathways and triggered osimertinib resistance.

**Conclusions:**

Intercellular transfer of exosomal wild type EGFR promotes osimertinib resistance in NSCLC, which may represent a novel resistant mechanism of osimertinib and provide a proof of concept for targeting exosomes to prevent and reverse the osimertinib resistance.

**Supplementary Information:**

The online version contains supplementary material available at 10.1186/s12943-021-01307-9.

## Background

Lung cancer is a leading cause of cancer-related deaths worldwide. NSCLC is the major histological subtype of lung cancer, which constitutes more than 85% of all lung cancer cases [1, 2]. While traditional cytotoxic chemotherapy remains an important treatment option for advanced NSCLC, recent advancement in personalized medicine allowed a subset of NSCLC patient harboring specific oncogenic mutations to respond well to targeted therapy with minimal adverse reaction. In particular, molecular targeted tyrosine kinase inhibitors (TKIs) specific to EGFR have been extensively studied [3]. Importantly, the mutations located in the *EGFR* gene were more frequently found in mainland China adenocarcinoma NSCLC patients (up to 50% ) [4]. NSCLC patients harboring these sensitizing EGFR mutations were exceptionally sensitive to the reversible first-generation EGFR TKIs (gefitinib and erlotinib) and the irreversible second-generation EGFR TKIs (afatinib and dacomitinib) [5, 6].

Despite the excellent initial clinical responsiveness and disease control rates, these patients inevitably developed acquired resistance to EGFR TKIs within an average of one year [7]. Among the various mechanisms leading to EGFR TKIs resistance, the gatekeeper T790M point mutation of EGFR close to the catalytic site is the most prevalent. EGFR T790M mutation is known to increase the affinity of the receptor tyrosine kinase for adenosine triphosphate (ATP) and also sterically hinder the binding of EGFR to TKIs, thereby leading to the failure of TKI treatment [8, 9]. Osimertinib is a novel mutant-selective irreversible third-generation EGFR TKI, which exhibits minimal effect on wild type EGFR (wtEGFR) and therefore less adverse effects, demonstrating potent anticancer activity in EGFR-mutated (mutEGFR) NSCLC patients harboring the T790M gatekeeper mutation [5]. After demonstrating robust objective response rate (ORR) and prolonging progression-free survival (PFS) in the AURA study (NCT01802632), osimertinib has been clinically approved as first-line treatment of advanced mutEGFR NSCLC patients regardless of T790M mutation status [10, 11]. While the discovery of osimertinib represents a breakthrough in the treatment of NSCLC, all patients eventually relapsed and developed resistance to the treatment. Importantly, there is no further effective therapeutic option for these progressing patients after failure of osimertinib.

Acquired resistance to osimertinib can be categorized into EGFR-dependent (such as EGFR C797S, G796D, G796S/R, L792F/Y/H, C797G, L718Q or L798I mutation, exon19 deletion and wild type *EGFR* gene amplification, or EGFR T790M loss) and EGFR-independent mechanisms (such as bypass pathway activation via MET or ERBB2, constitutive MAPK pathway activation by mutated KRAS, MEK or Src-AKT pathway activation ) [12–15]. Previous studies have showed that mutated *EGFR* gene arise somatically during tumorigenesis. Only a small sub-population of cancer cells carry the heterozygous activating EGFR mutations whereas others harbor the wild type counterparts [16, 17]. Recently, the relationship between intratumor heterogeneity and responsiveness to EGFR TKIs in NSCLC patients has been extensively studied, especially at the gene level [16, 18–20]. A higher percentage of cancer cells bearing the mutated *EGFR* gene within the tumor was highly correlated with better responsiveness, longer progression-free survival and overall survival in mutEGFR NSCLC patients. However, the specific mechanism underlying intratumor heterogeneity in EGFR mutation and osimertinib responsiveness remains unclear.

Exosomes (40–150 nm) are small extracellular vesicles originating from multivesicular bodies (MVBs) produced inside the endosomal compartment of most eukaryotic cells. They are released into the extracellular milieu upon the fusion of MVBs with plasma membranes. Exosomes act as mediators of cell-to-cell communication by delivering molecular constituents of cells (including nucleic acids and proteins) as their cargoes, thereby altering physiological state and biological functions of the recipient cells [21, 22]. Recently, exosomes have been shown to mediate the transfer of the drug resistance phenotype. Cancer cells would develop drug resistance after the incorporation of exosomes from drug-resistant cancer cells. Among these investigations, the transfer of microRNAs and long non-coding RNAs (lncRNAs) through exosomes is the most extensively studied [23–29]. In contrast, only a few recent studies reported about the acquisition and spread of drug resistance by protein cargoes delivered in exosomes [30–32].

In the clinic, the mutation status of *EGFR* gene in NSCLC patients is an important determinant of therapy decision [33]. The recent findings about the influence of intratumor heterogeneity on the responsiveness of tumor to EGFR TKIs were intriguing [34]. This study aims to investigate the possible intercellular transfer of wtEGFR protein via exosomes to mutEGFR NSCLC cells and subsequently activates PI3K/AKT and MAPK signaling pathways and triggers cell resistance to osimertinib. Moreover, the effect of osimertinib on exosomes formation and secretion and the pathway of exosomes uptake were also investigated, which provides novel insights in the field of osimertinib resistance induced by exosomes transfer.

## Materials and methods

### Chemicals and reagents

Osimertinib, gefitinib, afatinib, cisplatin and chlorpromazine (CPZ) were purchased from Selleck Chemicals (Houston, TX, USA). Osimertinib, gefitinib, afatinib and CPZ were prepared in dimethyl sulfoxide (DMSO) at a concentration of 10 mM or 100 mM whereas cisplatin was dissolved in dimethyl formamide (DMF). All stock solutions were kept at − 20 °C. Stock solutions were diluted to the appropriate concentrations with growth medium right before administration. Cetuximab was purchased from MedChemExpress (HY-P9905). PKH-67 and PKH-26 were purchased from Sigma-Aldrich (MINI67, MINI26). Generally, for in vivo experiment, phospho-ERK1/2 (T204), ERK1/2, phospho-AKT1/2/3 (S473) and AKT were purchased from Cell Signaling Technology (Danvers, MA, USA). For in vitro experiment, phospho-ERK1/2 (T204), ERK1/2, phospho-AKT1/2/3 (S473) and AKT were purchased from Santa Cruz Biotechnology (Dallas, TX, USA). CD63 and Alix antibodies were purchased from Santa Cruz Biotechnology (Dallas, TX, USA). EGFR and Caveolin-1 antibody were purchased from Cell Signaling Technology (Danvers, MA, USA). TSG101 and Calnexin antibodies were purchased from Affinity Biosciences (Cincinnati, OH, USA). RAB17 and GAPDH antibodies were purchased from Proteintech Group (Rosemont, IL, USA). Clathrin heavy chain antibody were purchased from Beyotime Biotechnology (Shanghai, CN). Polyclonal goat anti-mouse antibody and goat anti-rabbit antibodies were obtained from R&D systems (Minneapolis, MN, USA).

### Cell culture

H460, A549, H1299 (human NSCLC cell lines harboring wtEGFR), H1975 (human NSCLC cell line harboring L858R and T790M EGFR), PC9 (human NSCLC cell line harboring Exon 19del EGFR) and K562 (human EGFR-null chronic myeloid leukemia cell line) were cultured in DMEM medium (Gibco, USA) supplemented with 100 U/mL penicillin, 100 U/mL streptomycin and 10% fetal bovine serum (FBS) in a humidified incubator at 37 °C with 5% CO_2_. To eliminate exosomes from FBS used in cell culture, diluted FBS (1,4) was centrifuged at 150,000 *g* for 16 h at 4 °C in 70ti tube (Beckman Coulter OptimaTM L-100 XP).

### Exosomes isolation

Cancer cells were plated at a density of 10 million cells per T225 cm^2^ flask (Corning, USA) and cultured in DMEM contained 5% exosomes-depleted FBS for 48 h. Cancer cells-derived conditioned medium was pooled and exosomes were isolated by differential centrifugation according to previous published protocol with minor modifications [31]. Briefly, after removing cells and debris by at 300 *g* for 5 min and 2000 *g* for 25 min, the supernatant was harvested and centrifuged (Beckman Coulter Avanti J30I) at 15,000 *g* for 30 min to remove large extracellular vesicles. Finally, the supernatant was centrifuged at 100,000 *g* for 90 min (all centrifugation steps were performed at Beckman Coulter Avanti J30I at 4 °C). Exosomes were sedimented, re-suspended and washed in PBS followed by another ultracentrifugation procedure and the pellet was resuspended in 100 μL PBS.

For neutralizing assay, exosomes (20 μg) resolved in 1 mL PBS were incubated with cetuximab (5 μg/mL) at 4 °C for 2 h and then performed ultracentrifugation at 150,000 *g* for 2 h to remove excess antibody before incubated with H1975 cells or PC9 cells for 6 h.

For sucrose density gradients, pellet collected from ultracentrifugation (80 μg) was re-suspended in PBS, which was then overlaid with a linear sucrose gradient (10–70% w/v, pH 7.4) created by a gradient fractionator (Biocomp YIQI 113) in a SW41 tube (Beckman Coulter). The gradients were subjected to ultracentrifuge (Beckman Coulter OptimaTM L-100 XP) at 100,000 *g* for 16 h at 4 °C. Gradient fractions (1 mL) were collected from top to bottom and then the fractions were washed in PBS followed by ultracentrifugation (Beckman Coulter Avanti J30I) in JA30.50 tube at 100,000 *g* at 4 °C for 2 h. Pellets were directly lysed in RIPA buffer for further immunoblot analysis.

### Exosomes characterization

The exosome markers (TSG101, CD63, Alix) were used as positive control whereas the endoplasmic reticulum protein Calnexin was used as negative control in Western blot analysis. Number and size distribution of exosomes were analyzed by the Nanosight NS300 system (Nanosight Technology, Malvern, UK) according to manufacturer’s instructions. Exosomes resuspended in 50 μL PBS (pooled by 10 million cells for 48 h) were negatively stained with 2% uranyl acetate solution and imaged by JEM-1400 electron microscope (JEOL Ltd.,Japan), operated at 120 kV.

### Exosomes internalization analysis by flow cytometry

To calculate the internalization of exosomes, PC9 cells (1.5 × 10^5^/well) were seeded on 6-well plates. Exosomes (5 μg/well) were stained with PKH-67 according to manufacturer’s instructions and then added onto the PC9 cells under various condition. After incubation, cells were washed with PBS twice and subjected to analysis using flow cytometry.

### Immunofluorescence

To visualize the internalization of exosomes, PC9 cells (2 × 10^5^/mL) were seeded on 15 mm glass bottom cell culture disk (Nest, Cat#801002). Exosomes (5 μg/mL) were stained with PKH-26 according to manufacturer’s instructions and then added onto the PC9 cells under various condition. After pre-incubation for 12 h, cells were washed with PBS twice and fixed with 4% paraformaldehyde for 15 min at room temperature and stained with DAPI (5 μg/mL) for 15 min before imaging by ZEISS LSM880 confocal microscope.

To visualize the internalization of exosomal EGFR protein, H1975 cells (2 × 10^5^/mL) stably expressing RFP protein were seeded on 15 mm glass bottom cell culture disk (Nest, Cat#801002). Exosomes (25 μg/mL) collected from H1299 cells expressing EGFR-GFP fusion protein were added onto the H1975 cells. After pre-incubation for 6 h, cells were washed with PBS twice and fixed with 4% paraformaldehyde for 15 min at room temperature and stained with DAPI (5 μg/mL) for 15 min before imaging by ZEISS LSM880 or Nikon N-SIM confocal microscope. For visualization of EGFR and CD63 protein alteration, GFP or RFP-fusion protein were constructed accordingly for analysis. For visualization of the RAB17 and CD63 alteration after 36 h osimertinib treatment, cells were washed with PBS twice and fixed with 4% paraformaldehyde for 15 min at 25 °C followed by membrane permeabilization with 0.1% Triton X-100 for 10 min. Then cells were blocked with 3% BSA for 45 min and incubated at 4 °C with anti-CD63 mAb (1:250) and anti-RAB17 antibody (1:250) diluent in 1% BSA for 1 h. After incubating with secondary antibody conjugated with AlexaFluor 488 or AlexaFluor 568 (1:1000) for 45 min and staining with DAPI, cells were subjected to imaging by ZEISS LSM880.

### Measurement of mean fluorescence intensity from conditioned medium

To measure exosomes concentration, H1299 cells expressing CD63-GFP fusion protein was constructed and treated with 4 μM osimertinib for 36 h in phenol-free DMEM (Cat#21063029). After drug treatment, the supernatants were pooled and centrifuged at 15,000 g for 30 min to remove the dead cells, debris and large extracellular vesicles. Fluorescence intensity was then measured using the Tecan Spark fluorescence microplate reader.

### Construction and transfection of shRNA plasmid for RAB17, Caveolin-1, Clathrin and RAB27A knock-down

Lentiviral shRNA vectors (pLKO.1 puro) targeting RAB17 were constructed according to standard procedure and the genetic sequence was verified in US National Center for Biotechnology Information (NCBI). The shRNAs were co-transfected with psPAX2 and pMD.2G into 293 T cells at 70–80% confluence using Lipofectamine 2000 (Invitrogen) according to the manufacturer’s instructions. After 48 h, conditioned medium containing viral particles was pooled, filtered by 0.45 μm filter and cultured with H1299 cells or PC9 cells for 24 h at 37 °C. At 48 h after lentiviral infection, H1299 cells or PC9 cells were treated with puromycin (5 μg/mL). The changes in expression of the targeted proteins were determined by Western blotting analysis. The targeting sequences for specific genes were shown in Additional file: Table S1.

### Sanger sequencing of EGFR mutations

Genome DNAs of H460 cells, A549 cells, H1299 cells, H1975 cells and PC9 cells were extracted using DNA extraction kit (TIANGEN, Cat#DP304–03) according to the manufacturer’s instructions. The sequencing primers were shown in Additional file: Table S2.

### Western blot analysis

Expression of the proteins of interest was assessed by Western blot analysis, using the house-keeping protein GAPDH for normalization. Briefly, cells were lysed in RIPA buffer containing protease inhibitors and protein concentration was measured by BCA Protein Assay Kit (Pierce Biotechnology, USA). Cell lysates were electrophoresed in 10% SDS-PAGE gel and then transferred to a polyvinylidene difluoride (PVDF) membrane (Millipore, USA). After blocking by 5% BSA, membranes were probed with primary antibodies (1:1000) overnight at 4 °C and secondary antibody (1:5000) for 45 min at room temperature. Protein bands were visualized by ECL imaging (Pierce ECL kit, Thermo Fisher Scientific, USA).

### MTT assay

Cells pre-treated with various conditions were harvested during logarithmic phase and seeded onto 96-well plates at a density of 7000 cells/well in a final volume of 190 μL/well. After 12 h incubation, 10 μL of osimertinib, gefitinib, afatinib or cisplatin (indicated concentration) was added to 96-well plates. After 48 h treatment, 20 μL MTT solution (5 mg/mL) was added to each well and incubated for 4 h at 37 °C. Then, the supernatant was discarded and MTT crystal was dissolved in 100 μL DMSO. After incubating for 30 min, cell viability was measured by a microplate reader (Bio-Rad) at 570 nm (using 630 nm as reference wavelength). The 50% inhibitory concentration (IC_50_) was determined using the Bliss method. Experiments were performed at least three times.

### Apoptosis assay

Apoptosis assay was evaluated by Annexin V/PI apoptosis detection kit (BD Biosciences; San Jose, CA) according to the manufacturer’s instructions. Briefly, after treatment with 5 μM osimertinib for 36 h, cells were carefully harvested. After washing twice with PBS, cells were stained with Annexin V and PI diluent for 15 min and subjected to analysis using flow cytometry.

### Elisa

Briefly, H1299 cells were pre-treated with various concentration of osimertinib for 36 h, after which the supernatant was pooled and centrifuged at 15,000 *g* for 30 min to remove dead cells, debris and large extracellular vesicles. The expression levels of EGFR protein of the supernatants were measured by Human EGFR ELISA Kit (Solarbio, Cat#SEKH-0154) and the exosomes concentration was measured by Wako PS Capture™ exosome ELISA Kit (WAKO, Cat# 297–79,201) according to the manufacturers` instructions, respectively.

### Quantitative reverse transcription-PCR

Total cellular RNA was isolated by the Trizol reagent RNA extraction kit (Invitrogen, USA). Equal amount of RNA from different treatment groups was subjected to first-strain cDNA synthesis (*EZ*Bioscience, USA). Afterwards, real-time PCR was performed with Roche LightCycler 480. The expression of genes of interest in a given sample was normalized to GAPDH. The 2^-ΔΔCT^ method was applied to analyze the relative change in gene expression. The primers were listed in Additional file: Table S3.

### Colony formation assay

Cancer cells (2000 cells per well) were plated onto 6 well plates and treated with 1 μM osimertinib for 24 h. Next, culture medium was replaced with fresh DMEM containing 10% FBS and cultured for another 8 days (ectopic wtEGFR-expressing assay) or 14 days (co-culture assay). After that, the medium was removed and cells were washed with PBS twice, stained with 0.5% crystal violet, washed with tap water and then subjected to imaging and data analysis.

### Establishment of in vivo xenograft tumor models

In vivo experiments were conducted in accordance with the guidelines for the use of laboratory animals of the Sun Yat-Sen University Institutional Animal Care and Use Committee. H1975 cells (5 × 10^6^ cells) were subcutaneously injected into the right flank of athymic nude mice (BALB/c-nu/nu, female, 5 to 6 weeks old). When tumor volume reached 90 mm^3^, mice were randomized into three groups (seven in each group) and received different treatments: (a) Osimertinib (2–3 times/week, oral gavage, 5 mg/kg) plus exosomes (2–3 times/week, intratumor injection, 3 μg per mouse); (b) Osimertinib (2–3 times/week, oral gavage, 5 mg/kg) plus PBS (2–3 times/week, intratumorly); (c) Vehicle (5% DMSO+ 40% PEG300 + 5% Tween80 + 50% ddH_2_O, 2–3 times/week, oral gavage) plus PBS (2–3 times/week, intratumorly). Tumor size was measured with calipers every other day. Tumor volume was calculated by the formula: (length×width^2^/2). The mice were euthanized on day 29 and the xenografts were excised and weighed.

### Statistical analysis

The data were showed as means ± SEM and analyzed by one-way ANOVA or Student’s *t*-test analysis unless otherwise indicated. The statistical significance was determined to be *p* < 0.05. All statistical analyses were conducted by SPSS 22.0 or GraphPad Prism 7.

## Results

### Intercellular transfer of exosomal wtEGFR protein confers osimertinib resistance to sensitive mutEGFR cancer cells in vitro

Molecular intratumor heterogeneity is well recognized in NSCLC and the percentage of EGFR-mutated cancer cells has been reported to correlate well with the treatment response to EGFR TKI [20]. To verify the effects of NSCLC cells harboring wtEGFR on osimertinib-sensitive mutEGFR cancer cells proliferation and survival, NSCLC cells harboring wtEGFR (H460 cells, A549 cells, H1299 cells) and NSCLC cells with mutEGFR (H1975 cells, PC9 cells) were employed. The EGFR mutation status of four different NSCLC cells was confirmed by Sanger sequencing (Additional file: Fig. S1a) and their sensitivity to osimertinib was evaluated. As expected, H1975 cells and PC9 cells were much more sensitive to osimertinib than other NSCLC cells bearing wtEGFR indicated by MTT assay (Additional file: Fig. S1b).

**Fig. 1:**
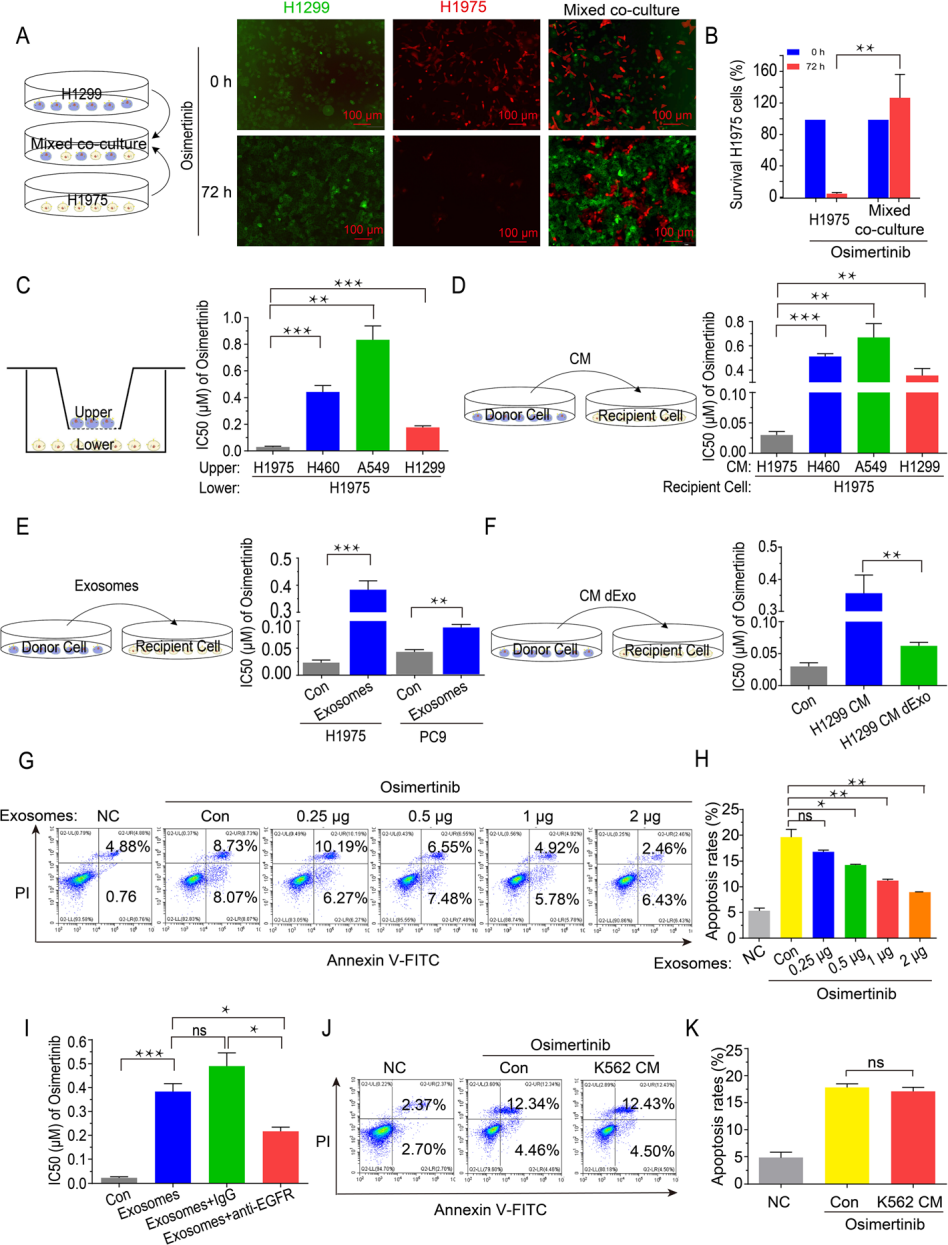
Intercellular transfer of exosomal wtEGFR protein confers osimertinib resistance to mutEGFR cancer cells in vitro. **a**-**b** Representative images of H1299 cells (GFP) alone, H1975 cells (RFP) alone or H1975 cells (RFP) co-cultured with H1299 cells (GFP) at a ratio of 1:1 treated by osimertinib (5 μM) for 72 h. Scale bar: 100 μm. **c** MTT assay of H1975 cells grown on the lower chamber indirectly pre-cultured with H460 cells, A549 cells and H1299 cells or control H1975 cells grown inside the transwell inserts (pores 0.4 μm) for 36 h. **d** MTT assay of H1975 cells pre-cultured with CM of H460 cells, A549 cells and H1299 cells or control H1975 cells for 36 h. **e** MTT assay of H1975 cells and PC9 cells pre-cultured with or without exosomes derived from H1299 cells for 6 h. **f** MTT assay of H1975 cells pre-cultured with CM or CM dExo derived from H1299 cells for 36 h. **g**-**h** Flow cytometric Annexin V/PI Apoptosis assay of H1975 cells pre-cultured with various concentration of exosomes for 6 h following osimertinib treatment (5 μM) for another 36 h. **i** MTT assay of H1975 cells pre-cultured with indicated exosomes (con, IgG or cetuximab pre-treated) derived from H1299 cells for 6 h. **j**-**k** Flow cytometric Annexin V/PI Apoptosis assay of H1975 cells pre-cultured with CM of K562 cells for 36 h following osimertinib treatment (5 μM) for another 36 h. All data are presented as means ± SEM. * *P* < 0.05, ** *P* < 0.01, *** *P* < 0.001

To examine the interaction between wtEGFR and mutEGFR cancer cells, we established H1299 cells stably expressing GFP and H1975 cells stably expressing RFP to perform direct co-culture assay. As shown, the survival of H1975 cells was dramatically increased upon osimertinib treatment after cocultivation with H1299 cells at a ratio of 1:1 (Fig. 1a and b). Furthermore, an in vitro transwell cell culture assay was performed to exclude the influence of direct contact between the different cell types on cell viability. Various cells (H460 cells, A549 cells, H1299 cells) were grown on the upper layer of a cell culture insert whereas the other cells (H1975 cells) were grown on the surface of the bottom chamber, so that the two cell types were separated by a transwell membrane (pores 0.4 μm). Compared with H1975 cells alone, the IC_50_ value of osimertinib was significantly increased in H1975 cells in the presence of upper layer H460, A549 or H1299 cells (Fig. 1c). Moreover, H1975 cells were also incubated with the pooled conditioned medium (CM) from NSCLC cells bearing wtEGFR (i.e., H460 cells, A549 cells, H1299 cells), after which cell viability (MTT and colony formation) and apoptosis assays were conducted following osimertinib treatment. Consistently, the presence of the CM from cancer cells bearing wtEGFR remarkably reduced the anti-proliferation and apoptotic effect induced by osimertinib in H1975 cells (Fig. 1d, Additional file: Fig. S1c-S1f).

To elucidate the mechanism by which wtEGFR-bearing NSCLC cells endowed sensitive mutEGFR H1975 cells with osimertinib resistance, we isolated exosomes from CM of H1299 cells and then performed MTT and apoptosis assay to validate. As expected, the IC_50_ value of osimertinib were increased significantly in H1975 cells and PC9 cells after pre-treatment with the exosomes derived from H1299 cells (Fig. 1e). Importantly, the osimertinib sensitiveness was rescued when exosomes were depleted from CM (CM dExo) by ultracentrifugation or inhibited by knockdown of RAB27A (Fig. 1f, Additional file: Fig. S1g-S1k), indicating the essential role of exosomes for the induction of osimertinib resistance. Moreover, it was noteworthy that apoptosis rates of H1975 cells following osimertinib treatment were reduced dramatically in a concentration-dependent manner after pre-treatment with various concentration of exosomes derived from H1299 cells (Fig. 1g and h).

Furthermore, to ascertain the functional role of wtEGFR protein in the observed osimertinib resistance, we applied cetuximab (5 μg/mL) as neutralized antibody to block exosomal wtEGFR and found that the osimertinib resistance phenotype induced by exosomes derived from H1299 cells was dramatically alleviated (Fig. 1i, Additional file: Fig. S1l). Moreover, the EGFR-null K562 cells were employed to further validate (Additional file: Fig. S1m and S1n). As shown, pre-treatment with CM of K562 cells did not alter the apoptosis rates of H1975 cells induced by osimertinib (Fig. 1j and k), implying the essential role of EGFR in the observed osimertinib resistance. Additionally, it was worthy to note when cisplatin was replacing osimertinib, there was ineligible difference between the responsiveness of H1975 cells to cisplatin in the presence or absence of supernatant from wtEGFR bearing NSCLC cells (Additional file: Fig. S1o). Collectively, intercellular transfer of exosomal wtEGFR protein was responsible for the induction of osimertinib resistance in sensitive mutEGFR cancer cells.

### Exosomal wtEGFR protein can be internalized by sensitive mutEGFR cancer cells via Clathrin-mediated pathway

**Fig. 2:**
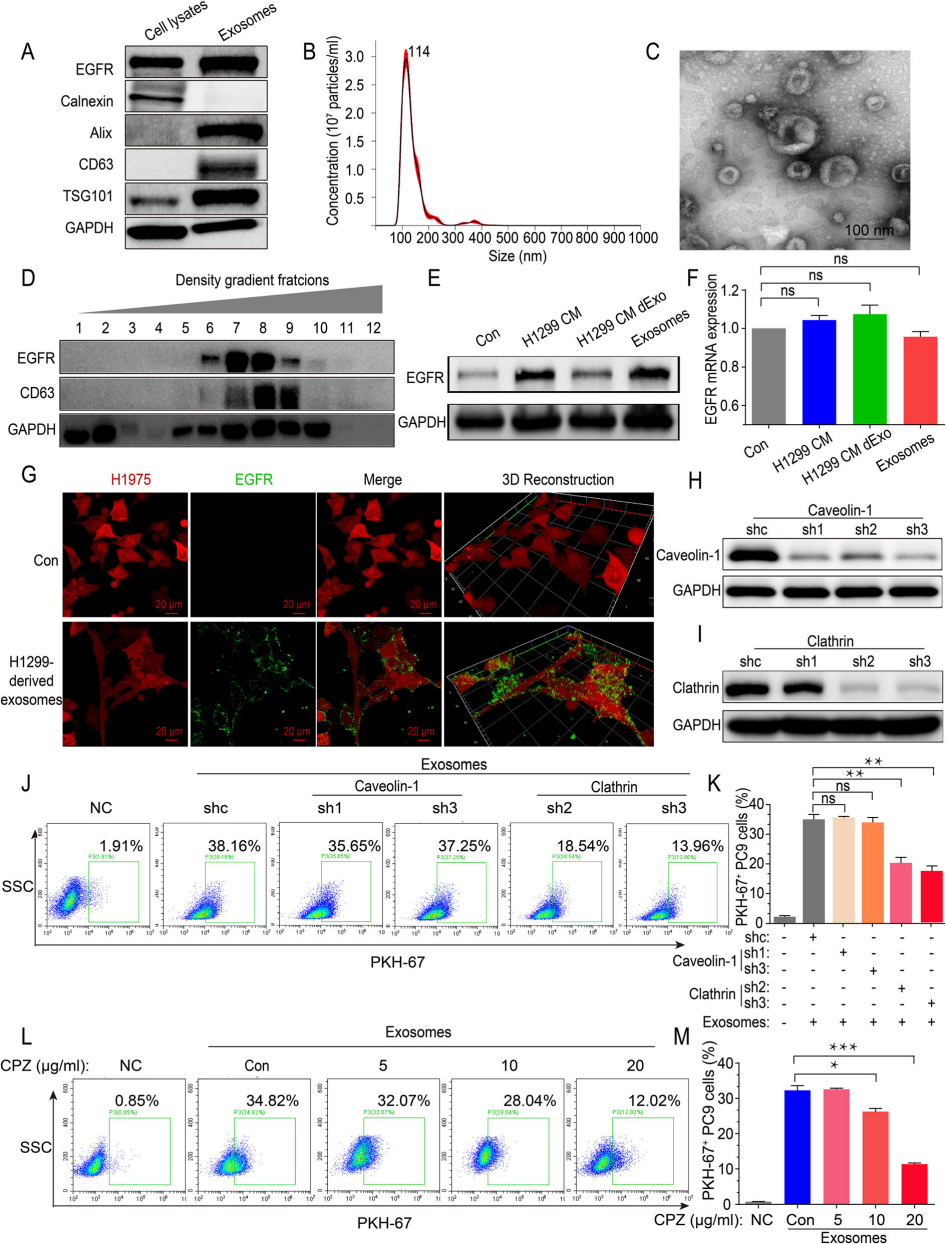
Exosomal wtEGFR protein can be internalized by sensitive mutEGFR cancer cells via Clathrin-mediated pathway. **a** Western blot analysis of indicated exosomal markers (positive markers: CD63, TSG101, Alix; negative marker: Calnexin) in H1299 whole cell lysates and H1299 cells-derived exosomes. **b** Nanoparticle Tracking Analysis of H1299 cells-derived exosomes. **c** Transmission Electron Microscope imaging of H1299 cells-derived exosomes. Scale bar: 100 nm. **d** Western blot analysis of colocalization of exosomal marker CD63 and transmembrane protein EGFR in various extracellular vesicle fractions as separated by sucrose density gradient centrifugation. **e** Western blot analysis of EGFR protein level of H1975 cells after pre-incubation with CM of H1299 cells, CM dExo of H1299 cells and H1299 cells-derived exosomes. **f** Real-time quantitative analysis of EGFR mRNA level of H1975 cells after pre-incubation with CM of H1299 cells, CM dExo of H1299 cells and H1299 cells-derived exosomes. **g** Representative images of internalization of H1299 cells-derived exosomes containing GFP-tagged wtEGFR in H1975 cells (RFP). Scale bar: 20 μm. (H) Western blot analysis of Caveolin-1 protein expression to verify the efficacy of Caveolin-1 silencing by shRNAs. **i** Western blot analysis of Clathrin protein expression to verify the efficacy of Clathrin silencing by shRNAs. **j**-**k** Flow cytometric exosomes absorption assay of control PC9 cells or Caveolin-1- and Clathrin-silenced counterparts treated with PKH-67-labeled exosomes (5 μg/mL) for 12 h. **l**-**m** Flow cytometric exosomes absorption assay of PC9 cells pre-cultured with various concentration of CPZ for 2 h following PKH-67-labeled exosomes (5 μg/mL) incubation for another 12 h. All data are presented as means ± SEM. * *P* < 0.05, ** *P* < 0.01, *** *P* < 0.001

To further confirm whether exosomal wtEGFR protein can be internalized by sensitive mutEGFR cancer cells, exosomes were isolated from CM of H1299 cells by differential ultracentrifugation and characterized by Western blot analysis (positive marker: CD63, TSG101, Alix; negative marker: Calnexin; as well as transmembrane protein of EGFR) (Fig. 2a), Nanoparticle Tracking Analysis (NTA) (Fig. 2b) and Transmission Electron Microscope (TEM) (Fig. 2c). Simultaneously, indicated by Western blot, EGFR protein was presented in exosomes of H460 cells, A549 cells and H1299 cells (Additional file: Fig. S2a). To further clarify whether EGFR protein was contained in exosomes, sucrose density gradient centrifugation was performed and found that EGFR was presented simultaneously with the exosome marker CD63 (Fig. 2d), suggesting that EGFR was preferentially encapsulated into exosomes.

To investigate whether exosomal wtEGFR can be delivered to the osimertinib-sensitive cancer cells, H1975 cells were cultured with the CM of H1299 cells or H1299 cells-derived exosomes and found that the EGFR protein was elevated without a significant change in EGFR mRNA expression. However, EGFR protein and mRNA level were not notably changed when H1975 cells were incubated with the CM of H1299 cells depleted of exosomes (Fig. 2e and f). Moreover, it was noteworthy that PKH-67-labeled H1299-derived exosomes could be quickly internalized by PC9 cells within 12 h (Additional file: Fig. S2b and S2c). Furthermore, in order to visualize the delivery of EGFR protein from H1299 cells to H1975 cells, an EGFR-GFP fusion protein lentivirus plasmid was engineered and transfected to H1299 cells. After RFP-tagged H1975 cells were incubated with these EGFR-GFP labeled H1299 cells-derived exosomes (25 μg/mL), green fluorescence from EGFR-GFP was integrated rapidly into the H1975 cells and could be detected on the cell membrane by confocal microscopy (Fig. 2g, Additional file: Fig. S2d,).

As known, there were two major endocytic pathways, a pathway associated with Caveolin-1 and another related to Clathrin, facilitating receptor internalization [31]. To further investigate by which pathway cancer cells absorb exosomes, we knocked down Caveolin-1 and Clathrin in PC9 cells using three shRNAs, respectively (Fig. 2h and i). As shown, the obvious decrease of Caveolin-1 did not decrease the internalization of exosomes in mutEGFR cancer cells, while knockdown of Clathrin contributed to a significant decrease of PKH-67-labeled cancer cells indicated by flow cytometry (Fig. 2j and k). Moreover, chlorpromazine (CPZ), a pharmacological inhibitor of Clathrin, could remarkably decrease the internalization of exosomes in a concentration-dependent manner (Fig. 2l and m, Additional file: Fig. S2e).

In conclusion, these results confirm that wtEGFR protein can be incorporated into exosomes and internalized by mutEGFR NSCLC cancer cells via Clathrin-mediated pathway.

### Intercellular transfer of exosomal wtEGFR protein activates PI3K/AKT and MAPK pathways in the presence of osimertinib in mutEGFR cancer cells

**Fig. 3:**
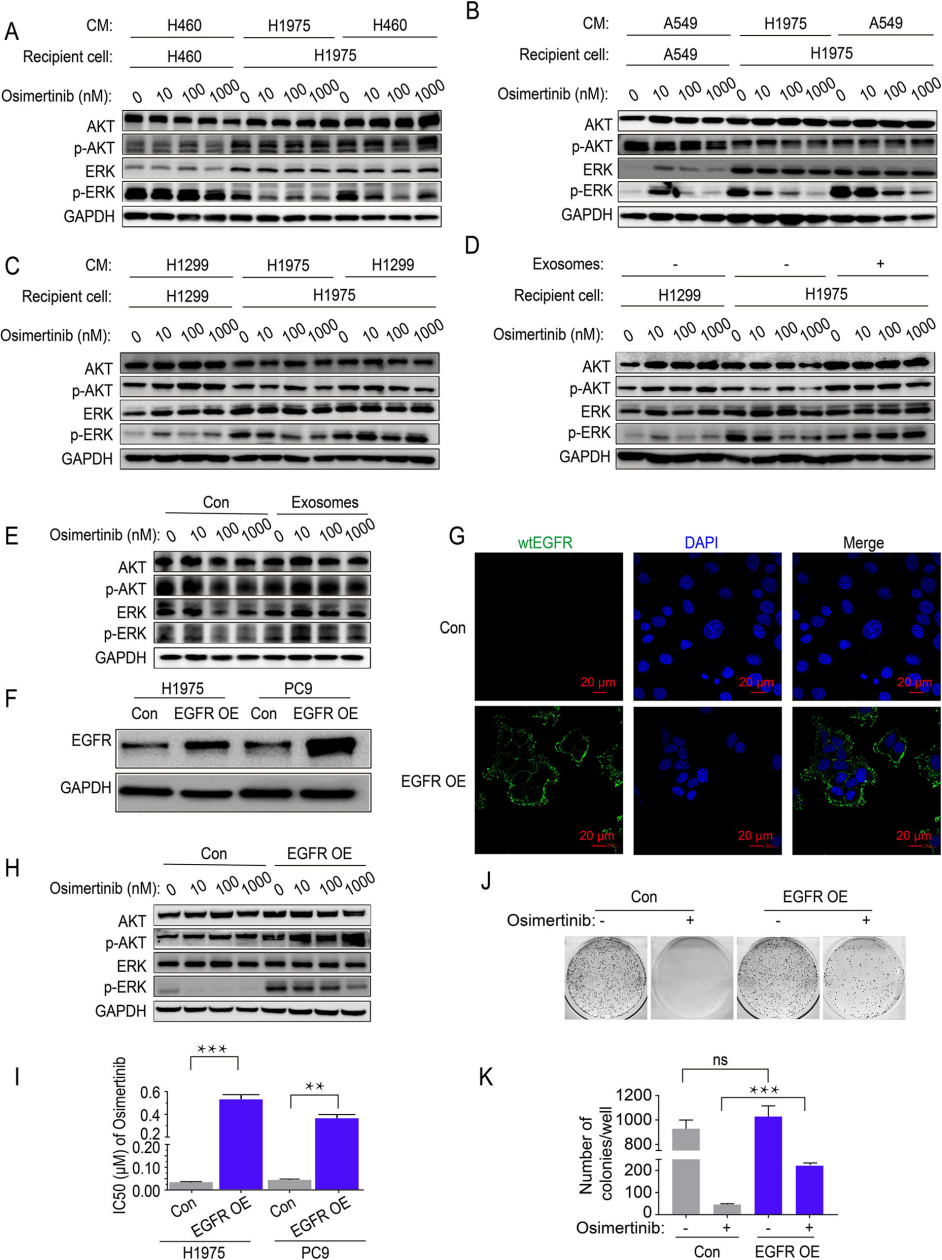
Intercellular transfer of exosomal wtEGFR protein activates PI3K/AKT and MAPK pathways in mutEGFR cancer cells. **a** Western blot analysis of indicated proteins in H1975 cells pre-treated with CM of H460 cells or control H1975 cells for 36 h and various concentration of osimertinib for another 6 h. **b** Western blot analysis of indicated proteins in H1975 cells pre-treated with CM of A549 cells or control H1975 cells for 36 h and various concentration of osimertinib for another 6 h. **c** Western blot analysis of indicated proteins in H1975 cells pre-treated with CM of H1299 cells or control H1975 cells for 36 h and various concentration of osimertinib for another 6 h. **d** Western blot analysis of indicated proteins in H1975 cells pre-treated with H1299 cells-derived exosomes or PBS for 6 h and various concentration of osimertinib for another 6 h. **e** Western blot analysis of indicated proteins in PC9 cells pre-treated with H1299 cells-derived exosomes or PBS for 6 h and various concentration of osimertinib for another 6 h. **f** Western blot analysis of EGFR protein in H1975 cells and PC9 cells with or without ectopic overexpression of wtEGFR protein. **g** Representative images of membrane-anchored wtEGFR (Flag-tag) in H1975 cells. Labels: blue, nucleus; green, wtEGFR (Flag-tag). Scale bar: 20 μm. **h** Western blot analysis of indicated proteins in H1975 cells treated with various concentration of osimertinib with or without ectopic overexpression of wtEGFR protein. **i** MTT assay of H1975 cells and PC9 cells with or without ectopic overexpression of wtEGFR protein. **j**-**k** Colony formation assay of H1975 cells with or without ectopic overexpression of wtEGFR protein after treated by osimertinib (1 μM) for 24 h. All data are presented as means ± SEM. * *P* < 0.05, ** *P* < 0.01, *** *P* < 0.001

To ascertain whether the intercellular transfer of exosomal wtEGFR protein activates EGFR downstream signaling pathway in mutEGFR cancer cells under osimertinib treatment, the status of the PI3K/AKT and MAPK pathways was evaluated after incubation with the CM from wtEGFR-bearing NSCLC cells. As expected, higher level of phosphorylation ERK (p-ERK) were observed after H1975 cells pre-incubated with CM of H460 cells, A549 cells or H1299 cells, while the level of p-AKT was slight elevated because of the limited inhibitory of osimertinib on p-AKT in control H1975 cells (Fig. 3A-3C). Additionally, the p-AKT and p-ERK were totally not blocked by osimertinib even up to 1000 nM in mutEGFR NSCLC cells pre-incubated with H1299 cells-derived exosomes whereas there were moderate inhibition on p-AKT and dramatic decrease of p-ERK in control mutEGFR NSCLC cells (Fig. 3 d and e).

More importantly, to further verify the effect of wtEGFR protein on mutEGFR cancer cells, we ectopically overexpressed wtEGFR (Flag-tag) in mutEGFR NSCLC cells and found that it was mainly localized in the plasma membrane confirmed by confocal microscopy (Fig. 3 f and g). Moreover, in H1975 cells ectopically overexpressing wtEGFR, the p-ERK was totally not inhibited by osimertinib and the level of p-AKT was much higher when compared with control H1975 cells (Fig. 3h). Further analysis revealed that ectopic overexpression of wtEGFR in H1975 cells and PC9 cells significantly increased cell survival and colony formation ability following osimertinib treatment (Fig. 3I-3K). Taken together, these results suggest that the exosomal wtEGFR might play an active role in activating PI3K/AKT and MAPK pathways in the presence of osimertinib in mutEGFR cancer cells.

### Osimertinib promotes the formation and secretion of exosomes in wtEGFR-expressing NSCLC cells

**Fig. 4:**
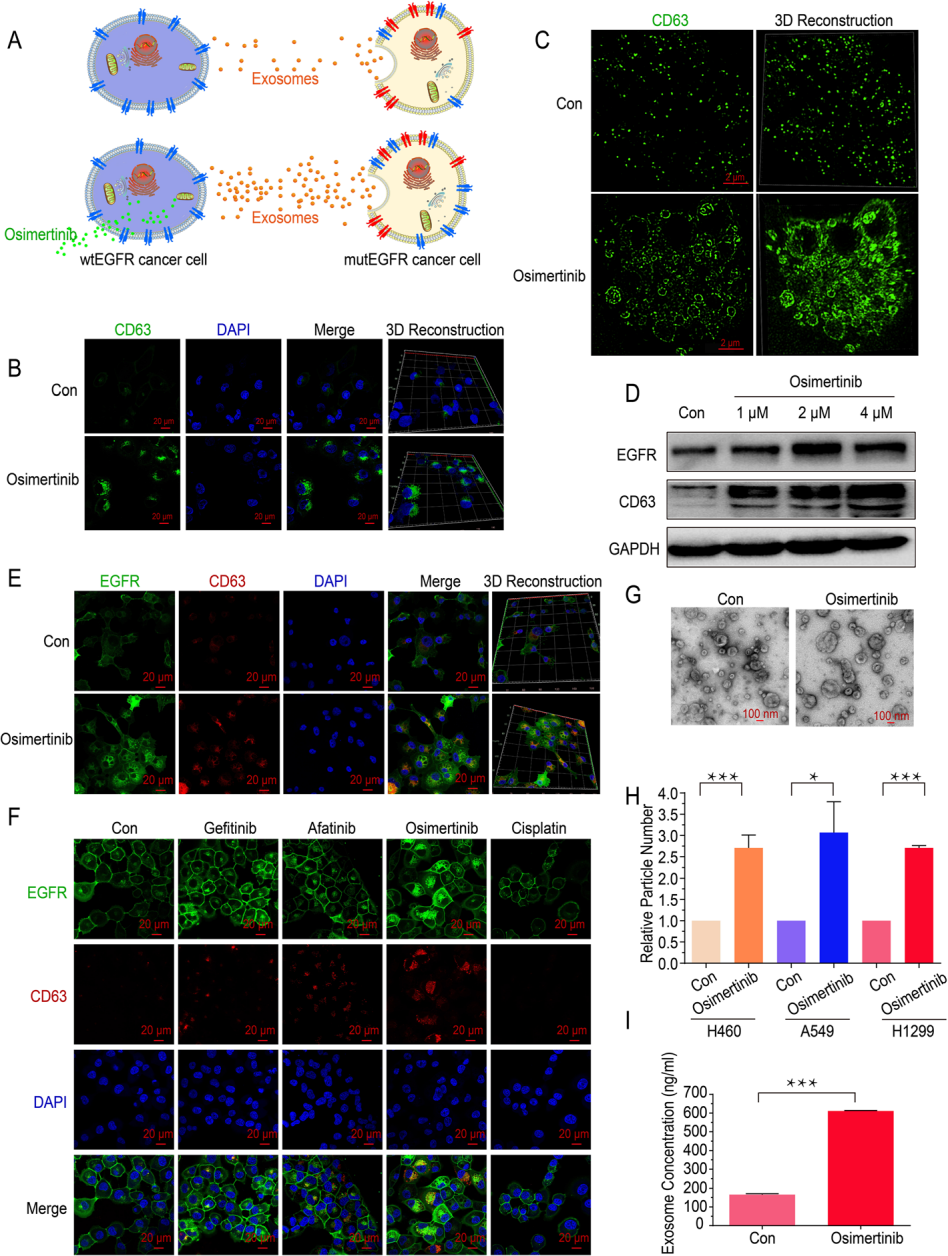
Osimertinib promotes the formation and secretion of exosomes in wtEGFR-expressing NSCLC cells. **a** Graphical scheme showing our working model of the promotion of exosomes release by osimertinib. **b** Representative images of cellular expression and localization of GFP-tagged CD63 in H1299 cells with or without osimertinib treatment (4 μM) for 12 h. DAPI is used for nucleus staining. Scale bar: 20 μm. **c** N-SIM imaging of cellular localization of GFP-tagged CD63 in H1299 cells with or without osimertinib treatment (4 μM) for 12 h. Scale bar: 2 μm. **d** Western blot analysis of indicated proteins level in H1299 cells treated by various concentration of osimertinib for 36 h. **e** Representative images of cellular expression and localization of GFP-tagged EGFR and RFP-tagged CD63 in H1299 cells with or without osimertinib treatment (4 μM) for 12 h. DAPI is used for nucleus staining. Scale bar: 20 μm. **f** Representative images of cellular expression and localization of GFP-tagged EGFR and RFP-tagged CD63 in H1299 cells treated by gefitinib (4 μM), afatinib (4 μM), osimertinib (4 μM) or cisplatin (50 μM) for 12 h. DAPI is used for nucleus staining. Scale bar: 20 μm. **g** Transmission Electron Microscope imaging of exosomes derived from H1299 cells with or without osimertinib treatment for 36 h. Scale bar: 100 nm. **h** Nanoparticle Tracking Analysis of exosomes in CM of H460 cells, A549 cells and H1299 cells after osimertinib treatment (4 μM) for 36 h. **i** Exosomes concentration in CM of H1299 cells after osimertinib treatment (4 μM) for 36 h measured by ELISA. All data are presented as means ± SEM. * *P* < 0.05, ** *P* < 0.01, *** *P* < 0.001

The data above demonstrates that exosomes from wtEGFR- expressing NSCLC cells result in osimertinib resistance in mutEGFR NSCLC cells. To further illustrate the mechanisms in detail, the effect of osimertinib on the formation and secretion of exosomes in NSCLC cells was also investigated (Fig. 4a). We first investigated the effect of osimertinib on the formation of exosomes in wtEGFR-expressing NSCLC cells. As shown, the classical exosome marker CD63 was remarkably increased in H1299 cells after treated with osimertinib (4 μM) (Fig. 4b). Furthermore, the location and quantified level of exosomes were assessed by Structured Illumination Microscopy (N-SIM) and found that the intracellular formation of exosomes dramatically increased in the presence of osimertinib compared with that in the absence of osimertinib in H1299 cells (Fig. 4c). Consistently, an increasing of CD63 protein level in wtEGFR-expressing NSCLC cells was observed in a concentration-dependent manner following osimertinib treatment indicated by Western blot (Fig. 4d, Additional file: Fig. S3a). In addition, it was noteworthy that we performed confocal imaging and found that EGFR expression was also increased and colocalized with exosomes in presence of osimertinib (Fig. 4e, Additional file: Fig. S3b). Likewise, to explore whether other anticancer drugs such as gefitinib, afatinib, and cisplatin also promote the formation of exosomes in wtEGFR-expressing NSCLC cells, confocal imaging was conducted and the results showed they did not significantly alter the formation of exosomes in H1299 cells (Fig. 4f).

Furthermore, to elucidate the effect of osimertinib on the secretion of exosomes, TEM was conducted and indicated no alteration in the morphology of secreted exosomes from H1299 cells after osimertinib treatment (Fig. 4g). In addition, we pooled cell culture supernatant of wtEGFR-expressing NSCLC cells and detected the number of small particles (presumably exosomes) by NTA. As expected, osimertinib dramatically promoted the secretion of exosomes (Fig. 4h). To further verify this, exosomes derived from the CM of H1299 cells were captured in Tim-4 immobilized plates and CD63 protein level was detected by ELISA as exosomes concentration. Consistently, there was a significant increase of secreted exosomes after osimertinib treatment (Fig. 4i). Hence, we speculate that osimertinib promotes the formation and secretion of exosomes in wtEGFR-expressing NSCLC cells.

### Osimertinib promotes the release of exosomes by upregulating RAB17

**Fig. 5:**
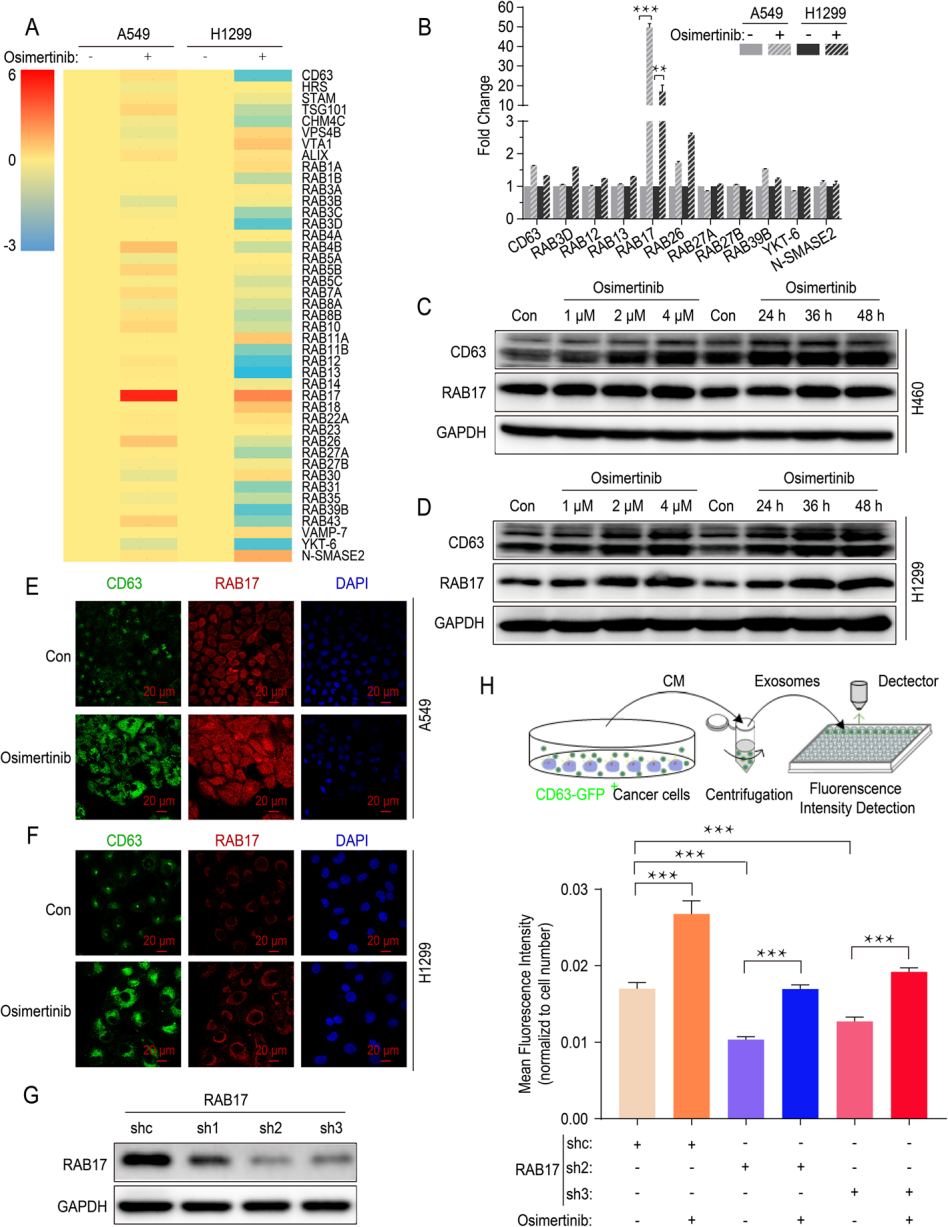
Osimertinib promotes the release of exosomes by upregulating RAB17. **a** Real-time quantitative PCR Screening for genes expression alteration encoding ESCRT or Rab GTPase family molecules in A549 cells and H1299 cells after osimertinib treatment (4 μM) for 12 h. Fold change in log2 transformation is shown as a color scheme. **b** Further validation of the changes in selected mRNAs in A549 cells and H1299 cells after osimertinib treatment (4 μM) by real-time quantitative PCR. **c** Western blot analysis of Rab17 and CD63 protein expression in H460 cells treated with various concentration of osimertinib for 36 h or treated with osimertinib (4 μM) for indicated time point. **d** Western blot analysis of Rab17 and CD63 protein expression in H1299 cells treated with various concentration of osimertinib for 36 h or treated with osimertinib (4 μM) for indicated time point. **e** Representative images of cellular expression and localization of RAB17 and CD63 protein in A549 cells after osimertinib treatment (4 μM) for 36 h. DAPI is used for nucleus staining. Scale bar: 20 μm. **f** Representative images of cellular expression and localization of RAB17 and CD63 protein in H1299 cells after osimertinib treatment (4 μM) for 36 h. DAPI is used for nucleus staining. Scale bar: 20 μm. **g** Western blot analysis of RAB17 protein expression to verify the efficacy of RAB17 silencing by shRNAs. **h** Mean Fluorescence Intensity of GFP (indicating CD63 expression) in CM of H1299 cells or RAB17-silenced counterparts after treated by osimertinib (4 μM). All data are presented as means ± SEM. * *P* < 0.05, ** *P* < 0.01, *** *P* < 0.001

To investigate the molecular mechanism that enhanced release of exosomes induced by osimertinib, quantitative real-time PCR was performed in A549 cells and H1299 cells to confirm the alterations of gene expression which were critical for endosome formation or exosomes secretion, including those encoding ESCRT proteins, Rab GTPase family and other related molecules (Fig. 5a). As shown, only RAB17 was identified as a key regulator in both A549 cells and H1299 cells exposed to osimertinib (Fig. 5b). The treatment of osimertinib led to a dramatic increase of RAB17 or CD63 protein level in a concentration- and time-dependent manner (Fig. 5c and d, Additional file: Fig. S3c). Moreover, it was worthy to note that an increase of RAB17 and CD63 protein level was simultaneously detected in A549 cells and H1299 cells by confocal microscopy (Fig. 5e and f).

To further confirm the critical role of RAB17 on the release of exosomes, RAB17 expression was silenced in H1299 cells using three different shRNAs and the efficient knock-down of RAB17 protein was verified by Western blot (Fig. 5g). In order to measure exosomes concentration more efficiently, a new fluorescence-based detection method was employed. Briefly, we established H1299 cells expressing CD63-GFP fusion protein (H1299 CD63-GFP^+^ cells) with or without RAB17 silencing. The CM from these CD63-GFP^+^ cells was collected and the GFP fluorescence intensity was measured after centrifugation. Using this fluorescence-based assay, we found that osimertinib significantly promoted the release of exosomes (Fig. 5g), which was consistent with previous results of NTA and ELISA (Fig. 4h and i). Importantly, when RAB17 expression was knocked down in H1299 cells by shRNAs, exosomes secreted by H1299 CD63-GFP^+^ cells were significantly reduced but this was rescued by osimertinib (Fig. 5h). Collectively, RAB17 is essential for the promotion of exosome release by osimertinib.

### Intercellular transfer of exosomal wtEGFR confers osimertinib resistance to mutEGFR H1975 xenograft in vivo

**Fig. 6:**
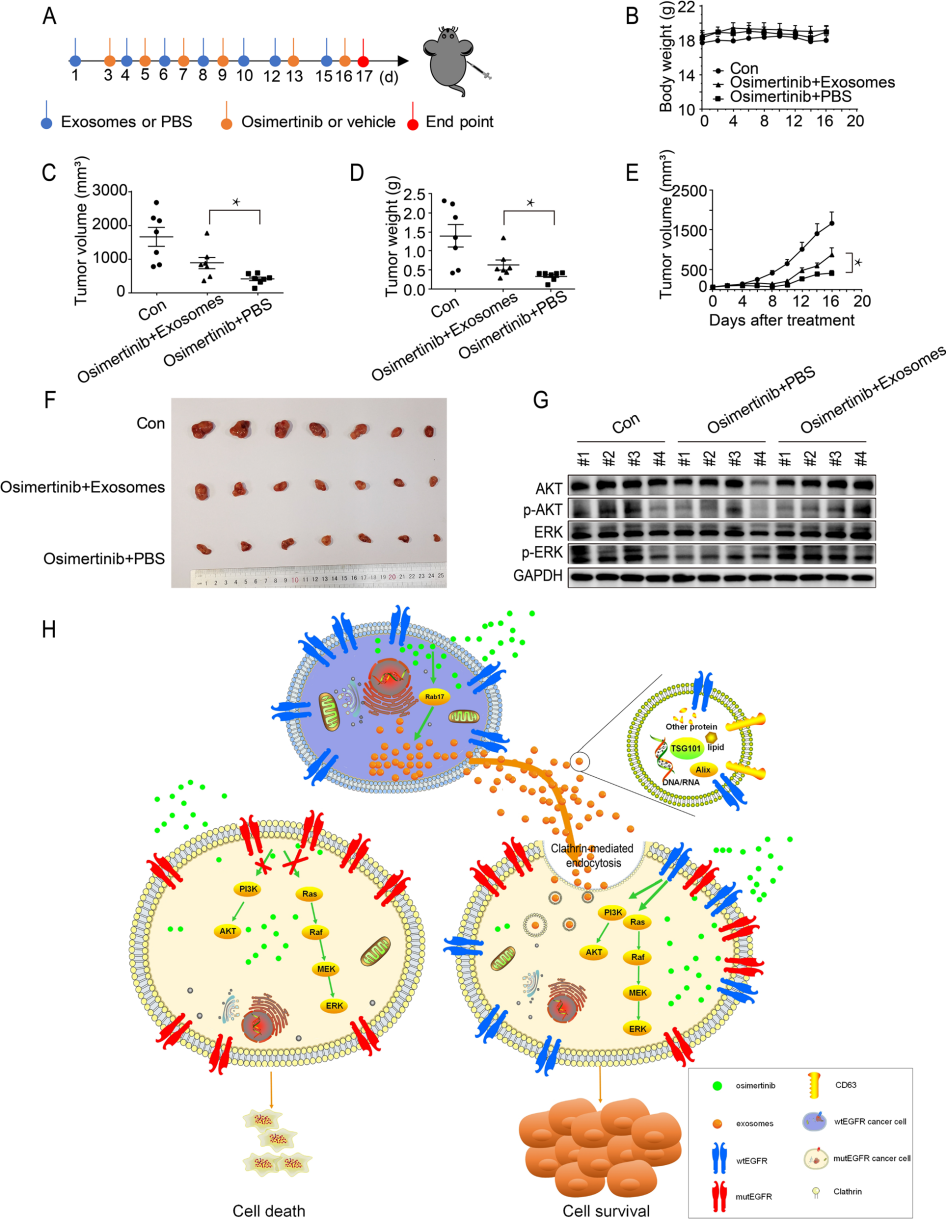
Intercellular transfer of exosomal wtEGFR confers osimertinib resistance to mutEGFR H1975 xenograft in vivo. **a** Schematic diagram depicting the in vivo experimental procedure. **b** Line graph showing the change in body weight of mice during experiment (*n* = 7). **c** Comparison of the tumor volume of three indicated treatment groups at end point (*n* = 7). **d** Comparison of the tumor weight of three indicated treatment groups at end point (*n* = 7). **e** Tumor growth curve at the indicated days with different treatments in BALB/c-nu/nu mice (*n* = 7). **f** Macroscopic view of tumor harvested at termination of three indicated treatment groups (*n* = 7). **g** Western blot analysis of indicated proteins level in xenograft tumors from three indicated treatment groups. **h** Working model showing the induction of osimertinib resistance by intercellular delivery of wtEGFR protein in exosomes from osimertinib-insensitive wtEGFR-expressing NSCLC cells to osimertinib-sensitive mutEGFR NSCLC cells. All data are presented as means ± SEM. * *P* < 0.05, ** *P* < 0.01, *** *P* < 0.001

To investigate the role of exosomes in osimertinib resistance in vivo, xenograft tumor model of mutEGFR H1975 cells was established in nude mice by subcutaneously injection of the cells into the right flank of the experimental animals. Different treatment interventions were carried out shown in scheme after palpable tumors have developed in the mice (Fig. 6a). H1299 cells-derived exosomes (3 μg) were injected into the H1975 xenograft tumors 2–3 times per week, which was followed by osimertinib (5 mg/kg) treatment at one day after exosomes injection. While osimertinib remarkably retarded tumor growth as expected, it is noteworthy that all treatment groups including the osimertinib+exosomes group did not cause appreciably animal body weight loss (Fig. 6b). At the end point of measurement (day 16), the osimertinib+exosomes group showed a larger tumor size and heavier tumor weight than the osimertinib+PBS group (Fig. 6c and d). Additionally, intratumor administration of H1299 cells-derived exosomes into the xenograft tumor significantly inhibited osimertinib-mediated reduction in tumor volume (Fig. 6e and f). Importantly, the activation status of the PI3K/AKT and MAPK pathways were also evaluated by Western blot. Consistent with the in vitro data, osimertinib alone effectively inhibited the p-AKT and p-ERK and the co-administration of osimertinib and H1299 cells-derived exosomes led to a considerable elevation of p-AKT and p-ERK (Fig. 6g). Collectively, these results demonstrate that exosomes derived from wtEGFR-expressing H1299 cells induce osimertinib resistance in mutEGFR H1975 cells by activating PI3K/AKT and MAPK pathways in vivo.

## Discussion

Osimertinib is administrated as first-line treatment in advanced NSCLC patients harboring mutEGFR [10, 11]. While the discovery of osimertinib represents a breakthrough in the treatment of NSCLC, almost all patients eventually relapsed and no further effective therapeutic options for the progressing patients. Mechanisms leading to osimertinib resistance are multifactorial and most investigations focused on genetic alteration of *EGFR* (in particular, *C797S*), and *MET* and *HER2* amplification [12, 13]. Recent researches showed that the status of intratumor heterogeneity was also responsible for osimertinib resistance. However, the specific mechanism remains unclear. In this study, we found osimertinib significantly promoted the formation and secretion of exosomes in wtEGFR-expressing NSCLC cells. These exosomes were enriched in wtEGFR protein and could be delivered to mutEGFR NSCLC cells, resulting in activation of PI3K/AKT and MAPK pathways under the stress of osimertinib. Mechanistically, osimertinib promoted the release of exosomes by upregulating RAB17 and then these exosomes could be internalized via Clathrin-mediated pathway. Figure 6h depicts a working model by which wtEGFR-incorporated exosomes play a novel role in mediating osimertinib resistance.

It is well recognized that tumors are inherently heterogeneous and their plastic phenotypes allow them to resist treatment [35, 36]. In an individual patient, it is noteworthy that just a part of cancer cells carries heterozygous activating mutations, whereas others harbor wild type counterparts. In 2011, Zhou et al. investigated the correlation between EGFR mutation abundance and clinical benefit from gefitinib treatment and found that median progression-free survival was significantly longer in patients with high abundances of EGFR mutations, indicating the relative EGFR mutation abundance could potentially predict clinal benefit from EGFR-TKI treatment. Moreover, a study conducted by Guo et al. analyzed 120 single tumor cells and confirmed the intratumor heterogeneity of EGFR-activating mutations in lung adenocarcinoma on the single-cell level, which might closely relate to EGFR-TKIs response in lung adenocarcinoma patients [16, 18–20]. Overall, the detection of EGFR mutation abundance is highly recommended in the clinic and could potentially predict EGFR-TKI response in NSCLC patients harboring mutEGFR.

Furthermore, NSCLC tumors bearing EGFR mutations are believed to express wtEGFR which could limit the clinical responsiveness and lead to shorter PFS upon osimertinib treatment. This hypothesis is partly supported by the findings that some NSCLC patients harboring EGFR mutation are refractory to osimertinib but they are responsive to erlotinib or afatinib [37, 38]. It has also been reported that NSCLC patients who developed drug resistance during osimertinib targeted therapy could be partially overcome by afatinib [39, 40]. Therefore, we hypothesized that selective pressure from osimertinib treatment allows tumor cells bearing wtEGFR to predominate and the cessation of osimertinib treatment may lead to the resurgence of mutEGFR. Consistently, a recent clinical trial conducted by Ichihara et al. demonstrated that re-administration of osimertinib to NSCLC previously showed acquired resistance could lead to tumor shrinkage once again with objective response and disease control rates of 33 and 73%, respectively [41]. Taken together, it is reasonable that the status of intratumor heterogeneity might partially determine the effect of osimertinib on mutEGFR NSCLC patients. Our study revealed that osimertinib-resistant wtEGFR-expressing NSCLC cells could secrete exosomal wtEGFR protein and deliver to osimertinib-sensitive cancer cells to activate the MAPK pathway, thus inducing osimertinib resistance. To this end, the co-administration of osimertinib and first- or second- generation EGFR TKIs might represent a useful strategy to overcome osimertinib resistance.

Exosomes are novel mediators of cell-to-cell communication to modulate cell signaling and biological function in recipient cells [22]. Recently, tumor-derived exosomes have been shown to mediate the transfer of drug resistance phenotype. Most researches in this area focused on the intercellular cross-talk by transferring miRNA or lncRNA from donor to recipient cells [23–29]. However, both miRNAs and lncRNAs can simultaneously affect multiple biological targets, which makes it difficult to predict their ultimate biological function and limits their clinical application. In NSCLC patients, the EGFR tyrosine kinase play a central role on tumorigenesis and the sensitivity to EGFR-targeted therapy [42]. Reported by several studies, EGFR protein could be incorporated into exosomes and delivered to the recipient cells to promote metastasis or enhance antiviral immunity [43–45]. In our study, we confirmed that exosomes carrying wtEGFR protein could be secreted from NSCLC cells harboring wtEGFR and incorporated into the cytomembrane of mutEGFR H1975 cells within several hours, thus inducing osimertinib resistance. Moreover, the depletion of exosomes from CM of H1299 cells, as well as the employment of neutralized body and CM of EGFR-null K562 cells, could not induce osimertinib resistance in H1975 cells, at least in part. Given that exosomes are known to be stable in various biological fluids, it is possible that the expression level of exosomal wtEGFR protein detected in plasma sample from NSCLC patients could be utilized to predict their responsiveness to osimertinib treatment. Indeed, normal cells and cancer cells would both secrete exosomes that containing EGFR (data not shown). The differences of these exosomal EGFR remain largely unexplored and it is a challenging work to define the origin of them. Further investigation about the clinical translation of our findings is warranted.

Inspired by previous report that chemotherapeutic drugs could promote the secretion of extracellular vesicles carrying the multidrug resistance transporter ABCB1 from multidrug resistant cancer cells and transfer to neighboring sensitive cells [31], the effect of osimertinib on exosomes biogenesis was also investigated in wtEGFR-expressing NSCLC cells in our study. As expected, we found that exosomes formation and secretion were dramatically increased in wtEGFR-expressing NSCLC cells following osimertinib (4 μM) treatment. Furthermore, screening aiming at molecules critical for endosome formation or exosomes secretion identified a member of the Rab GTPase family, RAB17, as the major contributor to the observed induction of exosomes formation and secretion by osimertinib. Unlike other Rab GTPases, RAB17 is expressed most abundantly in epithelial cells. It has been extensively studied in the delivery of hepatic transcytotic vesicles [46]. Recently, RAB17 has been identified as a tumor suppressor gene and low expression of RAB17 promotes the tumorigenesis via activation of the ERK pathway in hepatocellular carcinoma [47]. Honestly, our study is the first to report the regulation of formation and secretion of exosomes by RAB17 under exposure of osimertinib. Further studies are warranted to elucidate the mechanisms leading to the upregulation of RAB17 by osimertinib and the subsequent promotion of exosome secretion.

## Conclusions

In summary, our finding demonstrates that intercellular transfer of wtEGFR protein promotes osimertinib resistance by activating PI3K/AKT and MAPK signaling pathways both in vitro and in vivo. Moreover, osimertinib promotes the formation and secretion of exosomes by upregulating RAB17, which represents novel molecular target for possible circumvention of osimertinib resistance.

## Supplementary Information

**Additional file 1: Figure S1.** NSCLC cells harboring wtEGFR confer osimertinib resistance to sensitive mutEGFR cancer cells *in vitro.* **Figure S2.** Exosomal wtEGFR protein can be uptake by mutEGFR NSCLC cells via Clathrin. **Figure S3.** Osimertinib promotes the release of exosomes via RAB17. **Table S1.** shRNAs for RAB17, Caveolin-1, Clathrin and RAB27A. **Table S2.** Sequencing primers for EGFR mutation. **Table S3.** qPCR primers for screening and validation

## Data Availability

The datasets generated and/or analysed during the current study are available on the Research Data Deposit public platform (www.researchdata.org.cn). The approved RDD number is RDDB2021001047.

## Abbreviations

CPZ: Chlorpromazine
EGFR: Epidermal growth factor receptor
wtEGFR: Wild type EGFR
mutEGFR: EGFR-mutated
MVBs: Multivesicular bodies
N-SIM: Structured Illumination Microscopy
NSCLC: Non-small cell lung cancer
NTA: Nanoparticle Tracking Analysis
TEM: Transmission Electron Microscope
TKI: Tyrosine kinase inhibitors
