# Supplementary material for: Intercellular transfer of exosomal wild type EGFR triggers osimertinib resistance in non-small cell lung cancer
Source: Mol Cancer. 2021 Jan 18;20:17. doi: 10.1186/s12943-021-01307-9 (PMC7812728; doi:10.1186/s12943-021-01307-9)
Supplement: Supplementary file 1 — Additional file 1: Figure S1. NSCLC cells harboring wtEGFR confer osimertinib resistance to sensitive mutEGFR cancer cells in vitro. Figure S2. Exosomal wtEGFR protein can be uptake by mutEGFR NSCLC cells via Clathrin. Figure S3. Osimertinib promotes the release of exosomes via RAB17. Table S1. shRNAs for RAB17, Caveolin-1, Clathrin and RAB27A. Table S2. Sequencing primers for EGFR mutation. Table S3. qPCR primers for screening and validation [file 12943_2021_1307_MOESM1_ESM.zip › Additonal file-without tracked.docx]

**Figure legends**

**Figure S1. NSCLC cells harboring wtEGFR confer osimertinib resistance to sensitive mutEGFR cancer cells *in vitro*.**

(a) EGFR exon19, exon20 and exon21 sequence of H460 cells, A549 cells, H1299 cells, PC9 cells and H1975 cells were verified by Sanger sequencing. (b) IC_50_ of osimertinib in H460 cells, A549 cells, H1299 cells, PC9 cells or H1975 cells was measured by MTT assay. (c-d) Flow cytometric Annexin V/PI Apoptosis assay of H1975 cells pre-cultured with CM of H460 cells, A549 cells and H1299 cells or control H1975 cells for 36 h following osimertinib treatment (5 μM) for another 36 h. (e-f) Colony formation assay of H1975 cells pre-cultured with CM of H460 cells, A549 cells and H1299 cells or control H1975 cells for 36 h following osimertinib treatment (1 μM) for another 24 h. (g-h) Flow cytometric Annexin V/PI Apoptosis assay of H1975 cells pre-cultured with CM of H1299 cells, CM dExo of H1299 cells for 36 h or exosomes for 6 h following osimertinib treatment (5 μM) for another 36 h. (i) Western blot analysis of RAB27A protein expression to verify the efficacy of RAB27A silencing by shRNAs. (j) Nanoparticle Tracking Analysis of exosomes in CM of H1299 cells or RAB27A-silenced counterparts. (k) MTT assay of PC9 cells pre-cultured with CM derived from H1299 cells or RAB27A-silenced counterparts for 36 h. (l) MTT assay of PC9 cells pre-cultured with indicated exosomes (con, IgG or cetuximab pre-treated) derived from H1299 cells for 6 h. (m) Western blot analysis of EGFR protein level in K562 cells, H460 cells, A549 cells and H1299 cells. (n) EGFR protein level in CM of K562 cells, H460 cells, A549 cells or H1299 cells measured by ELISA. (o) IC_50_ of cisplatin by MTT assay in H1975 cells pre-cultured with CM of H460 cells, A549 cells and H1299 cells or control H1975 cells for 36 h. All data are presented as means ± SEM. * *P* < 0.05, ** *P* < 0.01, *** *P* < 0.001

**Figure S2. Exosomal wtEGFR protein can be uptake by mutEGFR NSCLC cells via Clathrin**

(a) Western blot analysis of exosomal marker CD63 and transmembrane protein EGFR in H460 cells, A549 cells and H1299 cells lysates and corresponding exosomes. (b-c) Flow cytometric exosomes absorption assay of PC9 cells cultured with PKH-67-labeled exosomes (5 μg/mL) at indicated time point. (d) N-SIM imaging of internalization of H1299 cells-derived exosomes containing GFP-tagged wtEGFR in H1975 cells (RFP). DAPI is used for nucleus staining. Scale bar: 2 μm. (e) Representative images of internalization of PKH-26-labeled H1299 cells-derived exosomes in PC9 cells with or without CPZ treatment (20 μg/mL) for 12 h. DAPI is used for nucleus staining. Scale bar: 20 μm. All data are presented as means ± SEM. * *P* < 0.05, ** *P* < 0.01, *** *P* < 0.001

**Figure S3. Osimertinib promotes the release of exosomes via RAB17.**

(a) Western blot analysis of CD63 protein expression in A549 cells treated with various concentration of osimertinib for 36 h. (b) Representative images of cellular expression and localization of RAB17 and CD63 protein in A549 cells after osimertinib treatment (4 μM) for 36 h. DAPI is used for nucleus staining. Scale bar: 20 μm. (c) Western blot analysis of Rab17 protein expression in A549 cells treated with various concentration of osimertinib for 36 h or treated with osimertinib (4 μM) for indicated time point.


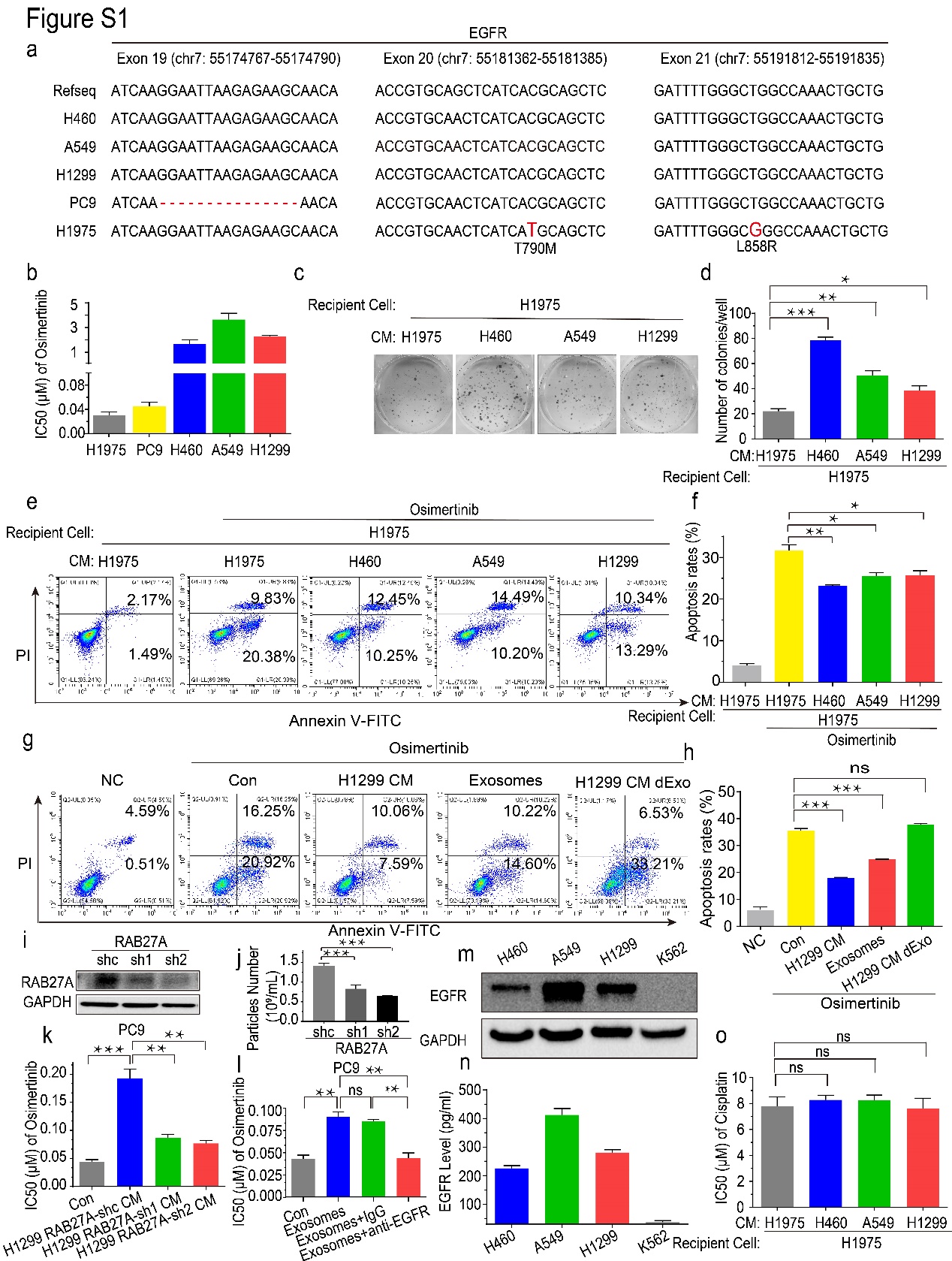

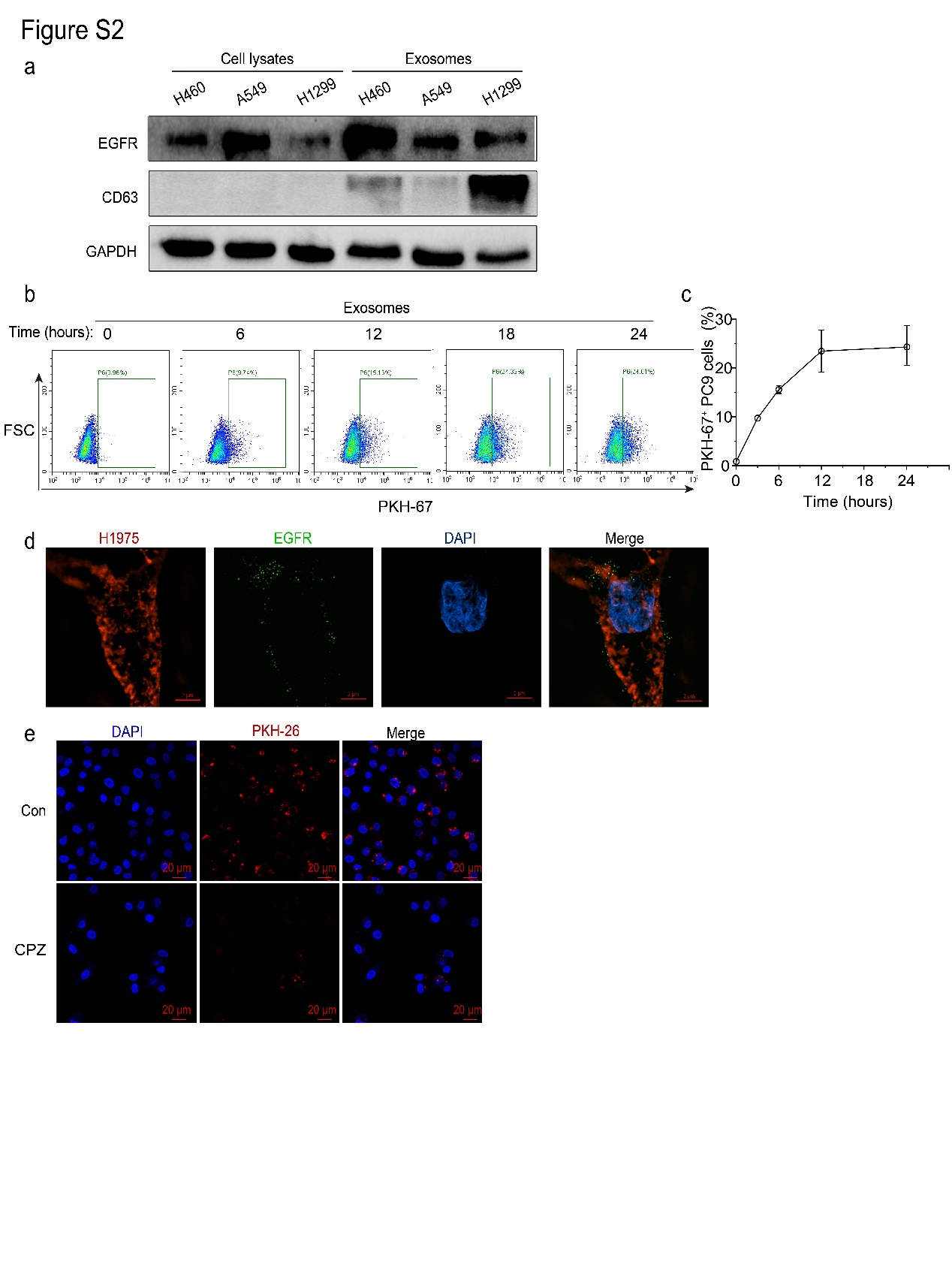

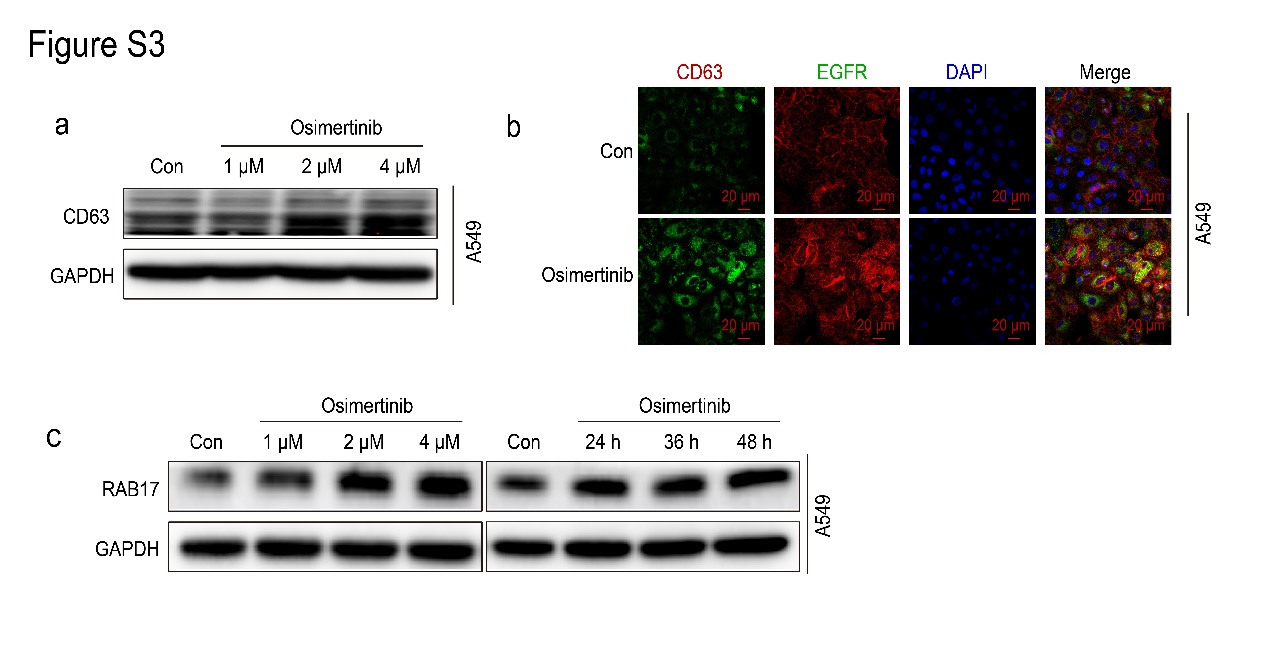


**Table S1. shRNAs for RAB17, Caveolin-1, Clathrin and RAB27A**

| Gene | Sequences |
| --- | --- |
| RAB17-sh1 | CCACCTCTCTGAAGCTTGA |
| RAB17-sh2 | CAGAAGTTGCTGTTCATGGAA |
| RAB17-sh3 | TCGGTACGTGAAGAACGACTT |
| Caveolin-1-sh1 | CCACCTTCACTGTGACGAAAT |
| Caveolin-1-sh2 | GCTTTGTGATTCAATCTGTAA |
| Caveolin-1-sh3 | GACGTGGTCAAGATTGACTTT |
| Clathrin-sh1 | CCAGAGAGATTTCTTCGTGAA |
| Clathrin-sh2 | GCCGACAAAGACAACACTAAT |
| Clathrin-sh3 | GCGAACATCAATAGATGCTTA |
| RAB27A-sh1 | CCAGTGTACTTTACCAATATA |
| RAB27A-sh2 | GCTGCCAATGGGACAAACATA |

**Table S2. Sequencing primers for EGFR mutation**

| Cell type | Exon primers | | Sequences |
| --- | --- | --- | --- |
| H460 | Exon 19-forward primer | TCAGTGTGATTCGTGGAGCC | |
|  | Exon 19-reverse primer | GGGGCTTAAACCCTATCCCAG | |
|  | Exon 20-forward primer | CCTCTCCCACTGCATCTGTC | |
|  | Exon 20-reverse primer | GTCCTGAATGGGGGAAGCAA | |
|  | Exon 21-forward primer | GTTAATGGTCAGCAGCGGGT | |
|  | Exon 21-reverse primer | TGCGCTATTGAAGGGGACTT | |
| A549 | Exon 19-forward primer | CCCATGGAATTGTCTTCAACTCTTA | |
|  | Exon 19-reverse primer | CGAGTGATCGTTTTGCTTGTCA | |
|  | Exon 20-forward primer | CTCTGCCTCATGGTCGTGTT | |
|  | Exon 20-reverse primer | AACCTGGTTTTGGCACCTCT | |
|  | Exon 21-forward primer | CAGCCATAAGTCCTCGACGTGG | |
|  | Exon 21-reverse primer | CATCCTCCCTGCATGTGTTAAAC | |
| H1299 | Exon 19-forward primer | TCAGTGTGATTCGTGGAGCC | |
|  | Exon 19-reverse primer | GGGGCTTAAACCCTATCCCAG | |
|  | Exon 20-forward primer | GACTCCGACTCCTCCTTTATC | |
|  | Exon 20-reverse primer | ATGGGACAGGCACTGATTT | |
|  | Exon 21-forward primer | GTTAATGGTCAGCAGCGGGT | |
|  | Exon 21-reverse primer | TGCGCTATTGAAGGGGACTT | |
| PC9 | Exon 19-forward primer | AGCAATATCAGCCTTAGGTG | |
|  | Exon 19-reverse primer | CATCATGCGTGTCAAGAAA | |
|  | Exon 20-forward primer | GACTCCGACTCCTCCTTTATC | |
|  | Exon 20-reverse primer | ATGGGACAGGCACTGATTT | |
|  | Exon 21-forward primer | TCAAGCCCAGGTCTCAACT | |
|  | Exon 21-reverse primer | CATTCACTGTCCCAGCAAG | |
| H1975 | Exon 19-forward primer | AGCAATATCAGCCTTAGGTG | |
|  | Exon 19-reverse primer | CATCATGCGTGTCAAGAAA | |
|  | Exon 20-forward primer | CTCTGCCTCATGGTCGTGTT | |
|  | Exon 20-reverse primer | AACCTGGTTTTGGCACCTCT | |
|  | Exon 21-forward primer | CAGCCATAAGTCCTCGACGTGG | |
|  | Exon 21-reverse primer | CATCCTCCCTGCATGTGTTAAAC | |

**Table S3. qPCR primers for screening and validation**

| Primers | Sequences |
| --- | --- |
| CD63-forward primer | CAACCACACTGCTTCGATCCTG |
| CD63-reverse primer | GACTCGGTTCTTCGACATGGAAG |
| HRS-forward primer | GACAGACTCTCAGCCCATTCCT |
| HRS-reverse primer | TCATGCGGTTCACGAAGGTGGT |
| STAM-forward primer | GACCAGTTGCTACAGATGCTGC |
| STAM-reverse primer | ATGAGAGGTCCCATCTGGTGAC |
| TSG101-forward primer | TTCTCAGCCTCCTGTGACCACT |
| TSG101-reverse primer | CCATTTCCTCCTTCATCCGCCA |
| CHM4C-forward primer | AGACTGAGGAGATGCTGGGCAA |
| CHM4C-reverse primer | TAGTGCCTGTAATGCAGCTCGC |
| VPS4B-forward primer | GGTTCTGGATTCTGCCATTAGGC |
| VPS4B-reverse primer | CCGAAAGTCTGCTTCCGTGAGA |
| VTA1-forward primer | GACCCAAGCAACATGCCATCAG |
| VTA1-reverse primer | GGCAGGTATAGTCTGTGGAGTAG |
| ALIX-forward primer | GCTCAGATGAGAGAAGCCACCA |
| ALIX-reverse primer | AGTCTGGATGCCTCCCTGTTCA |
| RAB1A-forward primer | GGGAACAAATGTGATCTGACCAC |
| RAB1A-reverse primer | GAAAGACTGTTCTACATTCGTTGC |
| RAB1B-forward primer | CCATCACTTCCAGCTACTACCG |
| RAB1B-reverse primer | TCGCTGGCATAGCGGTCAATCT |
| RAB3A-forward primer | CACCACCGCATACTACCGG |
| RAB3A-reverse primer | GTTTCCTACCAGCAGCACCTG |
| RAB3B-forward primer | CGCTATGCTGATGACACGTTCAC |
| RAB3B-reverse primer | CCCACGGTAATAGGCTGTTGTG |
| RAB3C-forward primer | GTCATCTCAACTGAGCGAGGTC |
| RAB3C-reverse primer | CACAAGGCGCTCAAATGTCTGC |
| RAB3D-forward primer | ACGTGTTGTGCCTGCTGAGGAT |
| RAB3D-reverse primer | CTTCTCGCAGATGACATCCACC |
| RAB4A-forward primer | ATGCAGGAACTGGCAAATCTTGC |
| RAB4A-reverse primer | CGTTCTTGTCCTGCTGTATCCC |
| RAB4B-forward primer | AATCGGCGTGGAGTTTGGAT |
| RAB4B-reverse primer | CCCCTCGGTAATAACTCCGC |
| RAB5A-forward primer | ACTTCTGGGAGAGTCCGCTGTT |
| RAB5A-reverse primer | GTGTCATCAAGACATACAGTTTGG |
| RAB5B-forward primer | GGAGACTTCAGCCAAGACAGCT |
| RAB5B-reverse primer | ACACTGGCTCTTGTTCTGCTGG |
| RAB5C-forward primer | AGTCTGCGGTAGGCAAATCCAG |
| RAB5C-reverse primer | TCATCCAGGCAGACAGTCTGTG |
| RAB7A-forward primer | GTGATGGTGGATGACAGGCTAG |
| RAB7A-reverse primer | AGTCTGCACCTCTGTAGAAGGC |
| RAB8A-forward primer | GATTCGCAACATTGAGGAGCAC |
| RAB8A-reverse primer | ATTGATGTTGGCCTTCGCGCT |
| RAB8B-forward primer | CAACACCACCTTCATCTCCACCA |
| RAB8B-reverse primer | GTTCGGAATCTTTCCTGACCCG |
| RAB10-forward primer | AAGGCGTTCCTCACGTTAGCTG |
| RAB10-reverse primer | GGAACAGGAGAATGCTCAGCAG |
| RAB11A-forward primer | AGCACCATTGGAGTAGAGTTTGC |
| RAB11A-reverse primer | AAGGCACCTACAGCTCCACGAT |
| RAB11B-forward primer | CAACTTGTCCTTCATCGAGAC |
| RAB11B-reverse primer | GATCTGTTTCTGTGACACGA |
| RAB12-forward primer | TGCGGTTCTGTGAAGCAAGTGC |
| RAB12-reverse primer | TTCTGGCGGTATCTCAGGCTCT |
| RAB13-forward primer | GACATCTTGCTCAAGTCAGGAGG |
| RAB13-reverse primer | CAGGGAGCACTTGTTGGTGTTC |
| RAB14-forward primer | GCGATTTAGGGCTGTTACACGG |
| RAB14-reverse primer | CCTTGCATCTGTCAACCAGCTG |
| RAB17-forward primer | ACAGCGTCTGCCACCTCTACTT |
| RAB17-reverse primer | CAGCATCACCAGGACTTCTCCT |
| RAB18-forward primer | CCTGCTCTTGAGGTTCACAGATG |
| RAB18-reverse primer | GTTCTAAACCTCTCTTGACCAGC |
| RAB22A-forward primer | GCACCAATGTACTATCGAGGGTC |
| RAB22A-reverse primer | CATGCTGTCGAAGCTCTTTCACC |
| RAB23-forward primer | GTAGCCGAAGTGGGAGATATACC |
| RAB23-reverse primer | ACCTTTTTGCCAGTGCCTCAGC |
| RAB26-forward primer | GCCTCCTTTGACAACATCCAGG |
| RAB26-reverse primer | TCCCTCTTCACCACACGCTCAT |
| RAB27A-forward primer | GAAGCCATAGCACTCGCAGAGA |
| RAB27A-reverse primer | CAGGACTTGTCCACACACCGTT |
| RAB27B-forward primer | TGGCAACAAGGCAGACCTACCA |
| RAB27B-reverse primer | CTCCACATTCTGTCCAGTTGCTG |
| RAB30-forward primer | GAAGCGCCAATGCCTTGATCCT |
| RAB30-reverse primer | CTCTCCTTTCAGCCAGGTCAATC |
| RAB31-forward primer | GGAGCTGAAAGAACATGGTCCAG |
| RAB31-reverse primer | GCACTTGTCTCAACCACGATGG |
| RAB35-forward primer | CAGCCCATCTTACTGCAAGCAG |
| RAB35-reverse primer | GCTGACAACCTGTCGGAGAGAA |
| RAB39B-forward primer | GAGCCAGGAAAACGCATCAAGC |
| RAB39B-reverse primer | GAAGACCACCTACTGAGTTCCTG |
| RAB43-forward primer | TGTCGGTGCCTCACTGGATTGA |
| RAB43-reverse primer | TGTCATAGTGCTCAGCCAGGCT |
| VAMP-7-forward primer | CGGTTCAAGAGCACAGACAGCA |
| VAMP-7-reverse primer | ATCCACTTGGGCTTGAGTCTCC |
| YKT-6-forward primer | CTGTTAGAGCGAGGTGAGAAGC |
| YKT-6-reverse primer | GATGGTGCCATTCCAGCATTGG |
| N-SMASE2-forward primer | AGAATCGTCGGGTACATCGC |
| N-SMASE2-reverse primer | GAAATCAGCCAGCCAGTCCT |
