# Supplementary figures and images for: Intercellular transfer of exosomal wild type EGFR triggers osimertinib resistance in non-small cell lung cancer
Source: Mol Cancer. 2021 Jan 18;20:17. doi: 10.1186/s12943-021-01307-9 (PMC7812728; doi:10.1186/s12943-021-01307-9)

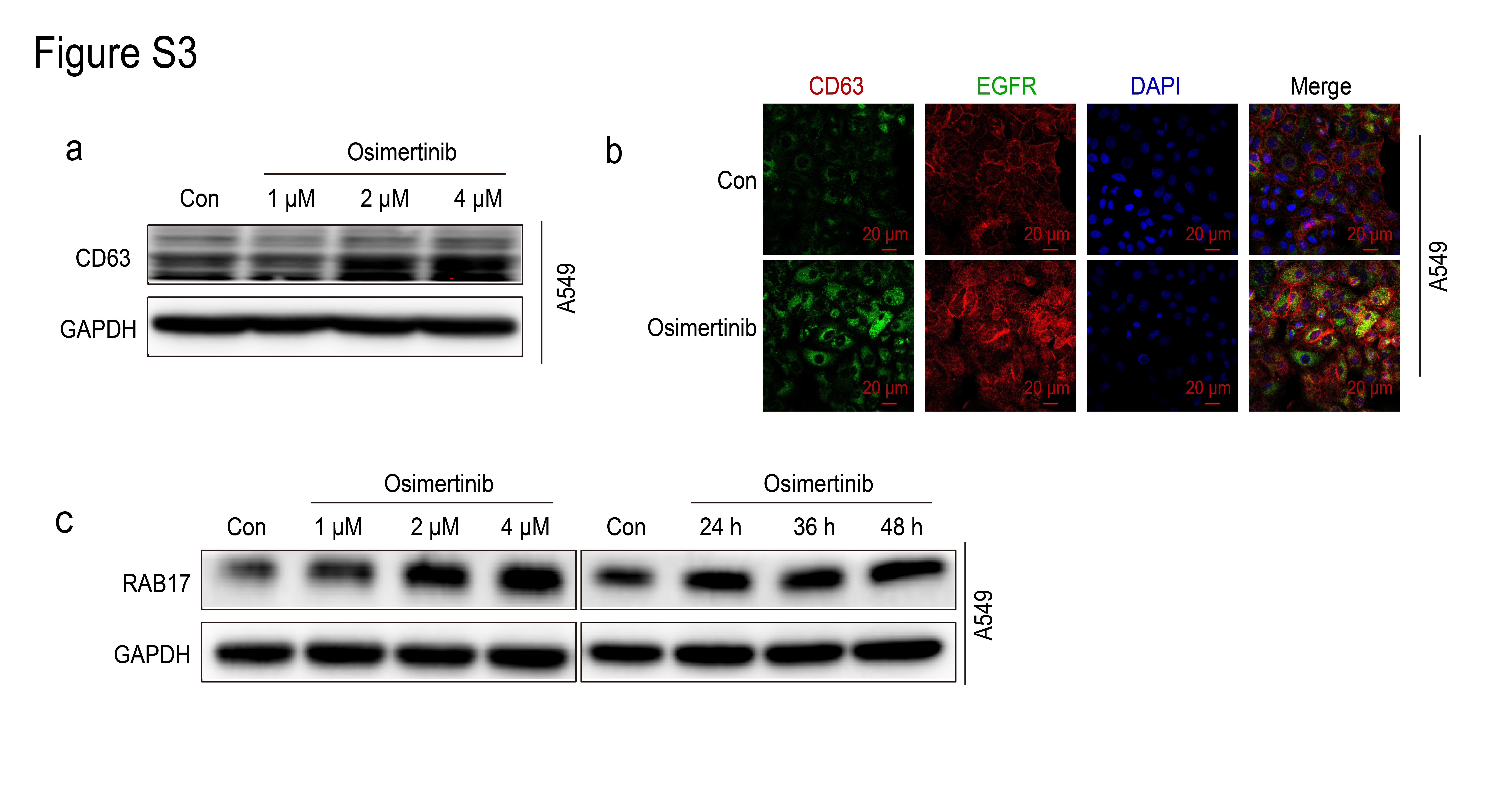

Supplement: Supplementary file 1 — Additional file 1: Figure S1. NSCLC cells harboring wtEGFR confer osimertinib resistance to sensitive mutEGFR cancer cells in vitro. Figure S2. Exosomal wtEGFR protein can be uptake by mutEGFR NSCLC cells via Clathrin. Figure S3. Osimertinib promotes the release of exosomes via RAB17. Table S1. shRNAs for RAB17, Caveolin-1, Clathrin and RAB27A. Table S2. Sequencing primers for EGFR mutation. Table S3. qPCR primers for screening and validation [file 12943_2021_1307_MOESM1_ESM.zip › Figure S3.jpg]
